# Structure of an N276-Dependent HIV-1 Neutralizing Antibody Targeting a Rare V5 Glycan Hole Adjacent to the CD4 Binding Site

**DOI:** 10.1128/JVI.01357-16

**Published:** 2016-10-28

**Authors:** Constantinos Kurt Wibmer, Jason Gorman, Colin S. Anthony, Nonhlanhla N. Mkhize, Aliaksandr Druz, Talita York, Stephen D. Schmidt, Phillip Labuschagne, Mark K. Louder, Robert T. Bailer, Salim S. Abdool Karim, John R. Mascola, Carolyn Williamson, Penny L. Moore, Peter D. Kwong, Lynn Morris

**Affiliations:** a Centre for HIV and STIs, National Institute for Communicable Diseases, National Health Laboratory Service, Johannesburg, South Africa; b Faculty of Health Sciences, University of the Witwatersrand, Johannesburg, South Africa; c Vaccine Research Center, National Institute of Allergy and Infectious Diseases, National Institutes of Health, Bethesda, Maryland, USA; d Institute of Infectious Disease and Molecular Medicine and Division of Medical Virology, University of Cape Town and National Health Laboratory Service, Cape Town, South Africa; e South African National Bioinformatics Institute, University of the Western Cape, Cape Town, South Africa; f Centre for the AIDS Programme of Research in South Africa, University of KwaZulu-Natal, Durban, South Africa; g Department of Epidemiology, Columbia University, New York, New York, USA; Ulm University Medical Center

## Abstract

All HIV-1-infected individuals develop strain-specific neutralizing antibodies to their infecting virus, which in some cases mature into broadly neutralizing antibodies. Defining the epitopes of strain-specific antibodies that overlap conserved sites of vulnerability might provide mechanistic insights into how broadly neutralizing antibodies arise. We previously described an HIV-1 clade C-infected donor, CAP257, who developed broadly neutralizing plasma antibodies targeting an N276 glycan-dependent epitope in the CD4 binding site. The initial CD4 binding site response potently neutralized the heterologous tier 2 clade B viral strain RHPA, which was used to design resurfaced gp120 antigens for single-B-cell sorting. Here we report the isolation and structural characterization of CAP257-RH1, an N276 glycan-dependent CD4 binding site antibody representative of the early CD4 binding site plasma response in donor CAP257. The cocrystal structure of CAP257-RH1 bound to RHPA gp120 revealed critical interactions with the N276 glycan, loop D, and V5, but not with aspartic acid 368, similarly to HJ16 and 179NC75. The CAP257-RH1 monoclonal antibody was derived from the immunoglobulin-variable IGHV3-33 and IGLV3-10 genes and neutralized RHPA but not the transmitted/founder virus from donor CAP257. Its narrow neutralization breadth was attributed to a binding angle that was incompatible with glycosylated V5 loops present in almost all HIV-1 strains, including the CAP257 transmitted/founder virus. Deep sequencing of autologous CAP257 viruses, however, revealed minority variants early in infection that lacked V5 glycans. These glycan-free V5 loops are unusual holes in the glycan shield that may have been necessary for initiating this N276 glycan-dependent CD4 binding site B-cell lineage.

**IMPORTANCE** The conserved CD4 binding site on gp120 is a major target for HIV-1 vaccine design, but key events in the elicitation and maturation of different antibody lineages to this site remain elusive. Studies have shown that strain-specific antibodies can evolve into broadly neutralizing antibodies or in some cases act as helper lineages. Therefore, characterizing the epitopes of strain-specific antibodies may help to inform the design of HIV-1 immunogens to elicit broadly neutralizing antibodies. In this study, we isolate a narrowly neutralizing N276 glycan-dependent antibody and use X-ray crystallography and viral deep sequencing to describe how gp120 lacking glycans in V5 might have elicited these early glycan-dependent CD4 binding site antibodies. These data highlight how glycan holes can play a role in the elicitation of B-cell lineages targeting the CD4 binding site.

## INTRODUCTION

Neutralizing antibodies to the HIV-1 envelope (Env) glycoprotein generally appear in all individuals within months of infection (1–4). These antibodies target highly sequence-variable epitopes that are fully accessible on prefusion Env trimers, such as the immunodominant, solvent-exposed, hypervariable regions V1 to V5 (2, 3, 5–8). As a result, these early neutralizing antibodies are strain specific for the transmitted/founder virus and rapidly select for escape mutants that drive Env diversification (6). Broadly neutralizing antibodies (bNAbs) that are able to cross-neutralize diverse HIV-1 strains by targeting structurally or functionally conserved regions of Env develop in some individuals later in infection (9–14). Animal studies have shown that bNAbs have the capacity to prevent infection and are likely the types of antibodies that will need to be elicited by an HIV-1 vaccine (15, 16). Significant effort has therefore gone into designing bNAb-initiating immunogens and understanding how bNAb precursors become broadly neutralizing. Studies defining the ontogeny of bNAbs have shown that they can develop from strain-specific precursors through affinity maturation, suggesting that in addition to recognizing hypervariable loop regions, strain-specific neutralizing antibodies might also overlap the conserved epitopes recognized by bNAbs (17–20). Furthermore, strain-specific or narrowly neutralizing antibodies have the potential to cooperate with other lineages in driving overall viral diversity, which in turn creates stimuli for the diversification of bNAbs (21, 22). Thus, studies of strain-specific antibodies are providing important insights for understanding how antibody lineages acquire neutralization breadth.

A large number of bNAbs targeting the CD4 binding site (CD4bs) have been isolated from HIV-1-infected individuals (18, 23–28). These antibodies can be adsorbed out of complex polyclonal sera by gp120 monomers, making them ideal candidates for isolation by flow cytometry. High-resolution crystal structures in complex with Env antigens have made this the most well-characterized site of vulnerability on the HIV-1 envelope (25, 26, 29). Two classes of CD4bs bNAbs have been described: the variable heavy (VH) gene-restricted class and the heavy chain complementarity-determining region 3 (CDR-H3)-dominated class. VH gene-restricted bNAbs all develop from the germ line-encoded immunoglobulin heavy chain variable gene IGHV1-2 or IGHV1-46 and were defined by prototypical antibodies VRC01 and 8ANC131 (25, 26, 29, 30). This class has a germ line-encoded arginine residue at position 71 in CDR-H2 that mimics an arginine at position 59 in CD4 by interacting with aspartic acid 368 in the CD4 binding loop of gp120. Over half of the VRC01 interaction with gp120 is mediated by CDR-H2 (30). As a result, VH gene-restricted CD4bs bNAbs are all similarly oriented with respect to Env. This angle of approach positions the light chains of IGVH1-2/46-derived CD4bs antibodies proximal to loop D in gp120, causing steric clashes with loop D and/or the glycan at position N276, which is thought to severely hamper the initiation of VH gene-restricted CD4bs bNAb lineages (31, 32). In contrast, members of the CDR-H3-dominated class of CD4bs antibodies are derived from a wider variety of IGHV genes and bind to Env with diverse angles of approach that are not impeded by the N276 glycan (26). Antibodies VRC13 (IGVH1-69), VRC16 (IGVH3-23), and CH103 (IGVH4-59) all form important interactions with D368 that develop through affinity maturation or germ line V-D-J recombination, similarly to VH1-2/46 class antibodies. A subgroup of the CDR-H3-dominated class, which includes HJ16 (IGVH3-30) and the more recently described antibody 179NC75 (IGVH3-21), has a different mode of recognition, where neutralization is independent of interactions with D368 but critically dependent on the N276 glycan (the same glycan that obstructs VH1-2/46 binding) (28, 33). Recombinant Env-derived proteins with a glycan deletion at position N276 are able to engage the germ line-reverted antibodies of many IGVH1-2/46-derived CD4bs bNAbs and have been proposed as HIV-1 vaccine immunogens (34, 35). Additional glycan deletions in V5 enable even better binding to VH gene-restricted bNAb precursors (32). These glycan deletions create large holes in the glycan shield, but their effects on Env antigenicity, and the immunogenicity of the CD4bs, remain to be determined.

We previously characterized the bNAb responses in plasma from donor CAP257, a participant of the CAPRISA cohort (36). Donor CAP257 developed three distinct waves of bNAbs that appeared sequentially in plasma, first targeting the V2 epitope, then targeting the CD4bs, and finally targeting a third, as-yet-undefined epitope. Donor CAP257 CD4bs bNAbs appeared between 50 and 60 weeks postinfection, with titers peaking at 122 weeks postinfection and declining thereafter. These bNAbs were critically dependent on the N276 glycan, and unlike most previously described CD4bs bNAbs, they could be readily adsorbed with D368R mutant gp120s. This CD4bs plasma bNAb response could be further divided into early and late CD4bs antibodies based on sensitivity to an N279D escape mutation that arose in the viral quasispecies at 93 weeks postinfection. Early CD4bs antibodies potently neutralized the tier 2 clade B strain RHPA but had very narrow neutralization breadth and were abrogated by the D279 immunotype. Late CD4bs antibodies were tolerant of the D279 immunotype, and this coincided with the acquisition of greater neutralization breadth. These broader antibodies were escaped first by mutations that removed the N276 glycan at 122 weeks postinfection and then by bulky aromatic side chain substitutions at position R456 in the base of V5 by 163 weeks postinfection.

Here, we designed RHPA-derived sorting antigens to isolate an early member of the CD4bs neutralizing antibody response, called CAP257-RH1, from donor CAP257 memory B cells. A cocrystal structure of CAP257-RH1 in complex with RHPA gp120 that included the N276 glycan revealed a CDR-H3-dominated interface that significantly overlapped the epitopes of CD4bs bNAbs. CAP257-RH1 neutralized one virus, RHPA, out of a large multiclade panel, and this heterologous strain specificity was explained by a binding angle that was incompatible with the V5 glycans present in most HIV-1 strains. Analysis of donor CAP257 autologous viruses by deep sequencing revealed a minority population with glycan-free V5 loops similar to those of RHPA, which may have initiated this lineage or provided the antigenic stimuli that allowed it to mature. These findings provide a mechanism for the strain specificity of early CD4bs neutralizing antibodies in donor CAP257, which target a rare glycan hole in V5 and have implications for the use of HIV-1 immunogens that are aimed at activating CD4bs bNAb precursors by removing key loop D and/or V5 loop glycans.

## MATERIALS AND METHODS

### Ethics statement.

The CAPRISA 002 Acute Infection cohort is comprised of women at high risk of HIV-1 infection in KwaZulu-Natal, South Africa. The CAPRISA Acute Infection study received ethical approval from the Universities of KwaZulu-Natal (E013/04), Cape Town (025/2004), and the Witwatersrand (MM040202). Donor CAP257 provided written informed consent for study participation. Blood samples were collected at regular intervals from seroconversion through to the initiation of antiretroviral therapy, processed, and cryopreserved individually.

### Cell culture and pseudovirus preparation.

293F suspension cells were cultured in 293Freestyle medium (Gibco BRL Life Technologies) at 37°C in 10% CO_2_ at 125 rpm. HEK293S (GntI^−/−^) cells were cultured in suspension as described above but supplemented with 2% heat-inactivated fetal bovine serum (FBS). Adherent CD4^+^ CCR5^+^ TZM-bl HeLa and HEK293T cells were grown to confluence in Dulbecco's modified Eagle's medium (DMEM) supplemented with 10% FBS, 22.7 mM HEPES (Gibco BRL Life Technologies), and 50 μg/ml gentamicin (Sigma) at 37°C in 5% CO_2_. Monolayers were disrupted with 0.25% trypsin in 1 mM EDTA (Sigma). For pseudoviruses, HEK293T cells were seeded in 10 ml at 2 × 10^6^ cells/ml in 10-cm^2^ dishes. Twenty-four hours later, plasmids expressing the Env protein of interest and the pSG3ΔEnv backbone (obtained from the NIH AIDS Research and Reference Reagent Program, Division of AIDS, NIAID, NIH) were cotransfected by using a 3:1 ratio of PEI-Max 40,000 (Polysciences). Cultures were incubated for 48 h at 37°C, filtered through a 0.45-μm filter, and frozen in 20% FBS. Mutant Env plasmids were made with the QuikChange Lightning kit (Stratagene).

### ELISA.

High-binding enzyme-linked immunosorbent assay (ELISA) plates were coated with recombinant gp120 at 4 μg/ml in Carb/Bicarb buffer at 4°C at pH 10 overnight. Plates were washed between each subsequent step four times with phosphate-buffered saline (PBS) containing 0.05% Tween 20. Blocking was done with 5% skim milk powder in PBS for 1 h at 37°C. Serial dilutions of HIV-1 monoclonal antibodies (MAbs) in 5% skim milk powder in PBS were added for 1 h at 37°C, and complexes were detected with an anti-Fc-horseradish peroxidase (HRP) conjugate. ELISA reactions were propagated in 100 μl of the enzyme substrate for 5 min and stopped with 25 μl of 1 M H_2_SO_4_. The absorbance was read at 450 nm.

### Neutralization assays.

Neutralization assays, performed as previously described (3, 37), were used to measure the reduction in relative light units after a single round of pseudovirus infection in the presence of a monoclonal antibody or plasma sample. Samples were serially diluted 1:3, and the 50% inhibitory dose (ID_50_)/50% inhibitory concentration (IC_50_) was calculated as the dilution at which infection was reduced by 50%.

### Adsorption assays.

Protein aliquots of 400 μg each were coupled to MyOne tosyl-activated magnetic Dynabeads (Invitrogen) at 37°C at pH 9.5 overnight and then blocked with 0.5% bovine serum albumin (BSA) in 0.0 5% Tween 20–PBS overnight at 37°C. Protein-conjugated beads were incubated with 200 μl of plasma (diluted 1:20) for 2 h at 37°C, the beads were then removed magnetically, and the remaining plasma was assessed for binding and neutralizing antibodies by using ELISAs and neutralization assays, respectively.

### B-cell sorting.

The positive RHPA-RC and negative RHPA-ADW gp120 antigens were engineered with a C-terminal AviTag, and the proteins were biotinylated postexpression by using a bulk BirA biotinylation kit (Avidity). Biotinylated sorting antigens were conjugated to streptavidin labeled with Alexa Fluor 647 (AF647) or brilliant violet 420 (BV420) and titrated for flow cytometry. Thawed peripheral blood mononuclear cells (PBMCs) were stained with sorting probes as well as the Live/Dead Aqua viability marker (Invitrogen, Carlsbad, CA), CD3-phycoerythrin (PE), CD14-PE, CD16-PE, IgD-fluorescein isothiocyanate (FITC), and CD19-allophycocyanin (APC)-Cy7 (BD Biosciences). Single sorted cells were frozen in RNase-free buffer. Flow cytometric data were acquired on a BD Aria 2 fluorescence-activated cell sorter (FACS), and the data were analyzed by using FlowJo (TreeStar).

### Immunoglobulin gene amplification.

Single B-cell heavy and light chain mRNA transcripts were reverse transcribed by using random hexamers and amplified by nested PCR in five different primer pools (three distinct sets for groups of variable heavy chain genes) as previously described (38, 39). Second-round PCRs introduced restriction sites that were used to clone the antibodies into mammalian expression vectors for IgG expression.

### X-ray protein crystallography.

The CAP257-RH1 monoclonal antibody containing a human rhinovirus (HRV) 3C protease cleavage site after the antigen binding fragment (Fab) was bound to protein A and used to purify an RHPA gp120 core that had already been enriched by Ni-nitrilotriacetic acid (NTA) chromatography (30 mM imidazole wash and 400 mM imidazole elution buffers at pH 7). The Fab-gp120 complexes were eluted with HRV3C and further purified by gel filtration using a Superdex 200 column (GE Healthcare). Complexes were concentrated to ∼8 mg/ml, aliquoted, and flash frozen in the presence of endoglycosidase H (Endo H) at ∼200 U/ml. Aliquots were thawed immediately before crystallization drops were set up. Two-hundred-nanoliter crystallization screens (containing 50% mother liquor) were set up under 576 conditions in 96-well sitting-drop vapor diffusion plates (Corning) at 25°C, using Cartesian Honeybee and TTP Labtech Mosquito crystallization robots. A single crystal hit was hand-optimized in 15-well hanging-drop diffusion plates using 1-μl drops at 25°C. The final crystallization conditions consisted of a solution containing 0.1 M HEPES (pH 7.5), 8% polyethylene glycol 4000 (PEG 4000), and 10% isopropanol, and crystals were flash frozen in 15% 2*R*,3*R*-butanediol or 25% 2,4-methylpentanediol as a cryoprotectant. Once mounted onto the goniometer, only 5% of the crystals yielded measurable X-ray diffraction, and this was considerably improved after these crystals were annealed *in situ* by diverting the cryostream for 3 s. Two of the crystals diffracted to a final resolution of 3.2 Å. Diffraction data were collected at the Advanced Photon Source (Argonne National Laboratory) SER-CAT ID-22 beamline, at a wavelength of 1.00 Å at 100K, and processed with HKL2000. The structure was solved by molecular replacement using the PHENIX v1.9-1692 software package and search models under PDB accession no. 4JZZ, 1NL0, and 4HPY. Models were refined with hydrogens to minimize clashes in COOT v0.8 using 5% of the data as an *R*_free_ cross-validation test set. All structural images were generated with the PyMOL Molecular Graphics System, Version 1.3r1edu (Schrodinger LLC).

### Next-generation sequencing library preparation.

RNA extraction, cDNA synthesis, and subsequent amplification were carried out as described previously (40), with the following modifications. A minimum of 5,000 HIV-1 RNA copies were isolated from longitudinal plasma samples spanning ∼3.5 years of infection by using the QIAamp viral RNA kit (Qiagen). The cDNA synthesis primer was designed to bind to the C5 region of the HIV-1 envelope gene (HXB2 gp160 DNA positions 1408 to 1431) and included a randomly assigned 9-mer tag (Primer ID method) to uniquely label each cDNA molecule, followed by a universal primer binding site to allow out-nested PCR amplification of cDNA templates. First-round amplification primers were designed to bind to the end of the V3 loop (HXB2 gp160 DNA positions 890 to 911) and contained a template-specific binding region, followed by a variable-length spacer of 0 to 3 randomly assigned bases to increase sample complexity. In addition, PCR primers contained 5′ overhangs, introducing binding sites for the Nextera XT indexing primers (Illumina). This allowed the amplification of the C3-to-V5 region of the envelope spanning HXB2 gp160 positions 911 to 1408. Nested PCRs were carried out by using the Nextera XT indexing kit. After indexing, samples were purified by using SPIRselect magnetic beads (Beckman Coulter) with a sample volume-to-bead ratio of 1:0.65 for the removal of <300-bp fragments. Sample concentrations were quantified by using the Qubit dsDNA HS assay (Thermo Fisher) and pooled in equimolar concentrations. The pooled amplicons were gel extracted by using the QIAquick gel purification kit (Qiagen) to ensure primer removal before the final library was submitted for sequencing on an Illumina MiSeq instrument, using 2-by-300-bp paired-end chemistry.

### Next-generation sequencing data processing and analysis.

Raw reads were processed by using a custom pipeline housed within the University of Cape Town High-Performance Computing core. Read quality was assessed by using fastqc (http://www.bioinformatics.babraham.ac.uk/projects/fastqc). Short reads (<150 bp) were filtered out by using Trimmomatic (41), and reads with an average quality score of less than 20 were removed by using a custom python script. A low *Q* score cutoff was used, as the Primer ID method removes sequencing and PCR errors during the consensus sequence generation step. The overlapping forward and reverse reads were merged by using PEAR (42), and the forward primer sequence was stripped out of all reads by using a custom python script. Reads where the forward primer could not be found were discarded. An in-house program, developed in R, was used to bin all reads containing an identical Primer ID tag, align the reads within each bin using MAFFT (43), and produce a consensus sequence based on a majority rule. Consensus sequences from bins of a size ≤*n* (where *n* is the maximum size of offspring bins expected to result from sequencing error in the PID of the largest bin and has a mean value of 15 for this data set) were filtered out by using a cutoff model described previously (44). Consensus sequences containing degenerate bases, frameshift mutations, or stop codons were also removed. Custom python scripts were used to calculate and plot the number and frequency of glycosylation sites in the V5 loop.

### Accession number(s).

The structure reported here is available under PDB accession no. 5T33.

## RESULTS

### Design of a resurfaced gp120 core sorting antigen based on tier 2 strain RHPA.

**FIG 1:**
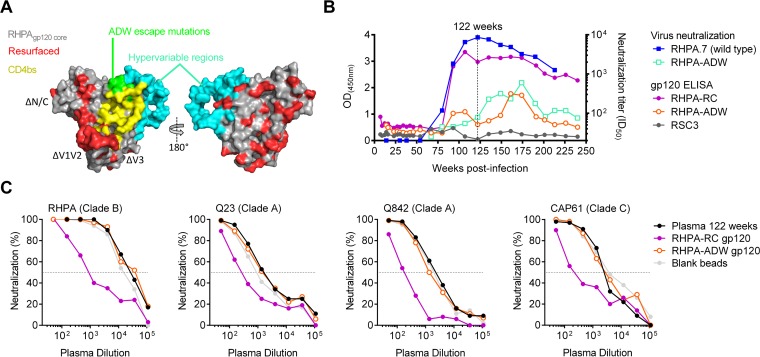
Resurfaced RHPA gp120 antigens bind differentially to CAP257 CD4bs plasma antibodies. (A) Surface view of a modeled RHPA gp120 core showing the location of N/C-terminal truncations; deleted V1, V2, or V3 loops; resurfacing mutations; and the N276D/T278A, N279D, and R456W escape mutations, relative to the CD4 binding site. Hypervariable regions α2, β14, V4, and V5 were not resurfaced. (B) Comparison of the abilities of longitudinal donor CAP257 plasma samples to bind to the newly designed gp120 core sorting antigens RHPA-RC and RHPA-ADW by ELISAs and neutralization of the heterologous tier 2 strain RHPA or the RHPA-ADW mutant variant resistant to CAP257 CD4bs antibodies. The lack of binding to RSC3 is shown in gray. Absorbance readings are plotted on the left *y* axis, neutralization titers are plotted on the right *y* axis, and time course postinfection is plotted on the *x* axis. The vertical dotted line indicates the 122-week time point at which the adsorption experiments described below for panel C were performed. (C) Adsorption of CAP257 peak CD4bs neutralizing titers by the positive gp120 antigen RHPA-RC but not the negative gp120 antigen RHPA-ADW or uncoated beads. Neutralization of four HIV-1 strains by donor CAP257 plasma that had been adsorbed with blank beads or beads coated with the positive gp120 antigen RHPA-RC or the negative gp120 antigen RHPA-ADW is plotted on the *y* axis as percent inhibition. Serial dilutions of the adsorbed plasma are indicated on the *x* axis.

We have previously shown that the CD4bs neutralizing antibodies in plasma from donor CAP257 were similar to HJ16 (36) and could not be adsorbed with the resurfaced stabilized gp120 core (RSC3) sorting antigen used to isolate VRC01. Therefore, a new sorting antigen was engineered for donor CAP257 B-cell isolation based on tier 2 strain RHPA, which was highly sensitive (ID_50_ titer of ∼1:8,000) to the CD4bs antibodies of donor CAP257. Similarly to RSC3, the RHPA gp120 sorting antigens were truncated in the V1-V3 loops as well as the N and C termini and resurfaced with 47 rare mutations in surface-exposed but normally conserved residues outside the CD4bs (Fig. 1A; see also Fig. S1A in the supplemental material) (24). Hypervariable regions are unlikely to be reactive with heterologous antibodies and were not resurfaced. The RSC3 cavity-filling mutations (M95W, T257S, S375W, and A433M) and cystine bonds near loop D (W96C/V275C) that were previously designed to stabilize gp120 in the CD4-bound conformation were avoided, because it was not known how they might affect the CAP257 epitope, which differed from VRC01-like antibodies (36). This resurfaced core gp120, called RHPA-RC, bound to conformation-sensitive neutralizing antibodies VRC01 and 2G12, confirming that resurfacing mutations did not compromise protein folding (data not shown). To enhance the specificity for CAP257 CD4bs antibodies, we also created a negative sorting antigen, called RHPA-ADW, which included three mutations in gp120 loop D and the base of V5 (T278A, N279D, and R456W) that we have previously shown to contribute to escape from the CD4bs neutralizing antibodies in donor CAP257 plasma (Fig. 1A, green) (36). The negative antigen RHPA-ADW did not bind strongly to CD4bs antibody VRC01 as expected but still bound well to 2G12, confirming the antigen's conformational integrity and its ability to select for CD4bs antibody responses (data not shown).

The ability of CAP257 CD4bs antibodies to bind to either the positive RHPA-RC antigen or the negative RHPA-ADW antigen was assessed by ELISAs and adsorption assays. Remarkably, the longitudinal kinetics of CAP257 plasma antibody binding to the positive antigen RHPA-RC tracked with the neutralization of the RHPA parent strain, with titers increasing or decreasing at similar time points throughout infection (Fig. 1B). Similarly, CAP257 plasma antibodies did not bind well to the negative antigen RHPA-ADW, although they showed weak binding at between 161 and 174 weeks postinfection that tracked with the neutralization of the more resistant RHPA-ADW mutant virus (Fig. 1B). Adsorption experiments using donor CAP257 plasma at 122 weeks postinfection, when the CD4bs antibody titers had peaked, showed that the positive antigen RHPA-RC could efficiently adsorb the CD4bs neutralizing antibodies against RHPA (a clade B virus), Q23 and Q842 (clade A), and CAP61 (clade C), while the negative antigen RHPA-ADW could not (Fig. 1C). Altogether, these data suggested highly specific selection for CAP257 CD4bs neutralizing antibodies by the combination of the positive RHPA-RC and negative RHPA-ADW sorting probes.

### Isolation of an N276 glycan-dependent CD4bs B-cell lineage.

**FIG 2:**
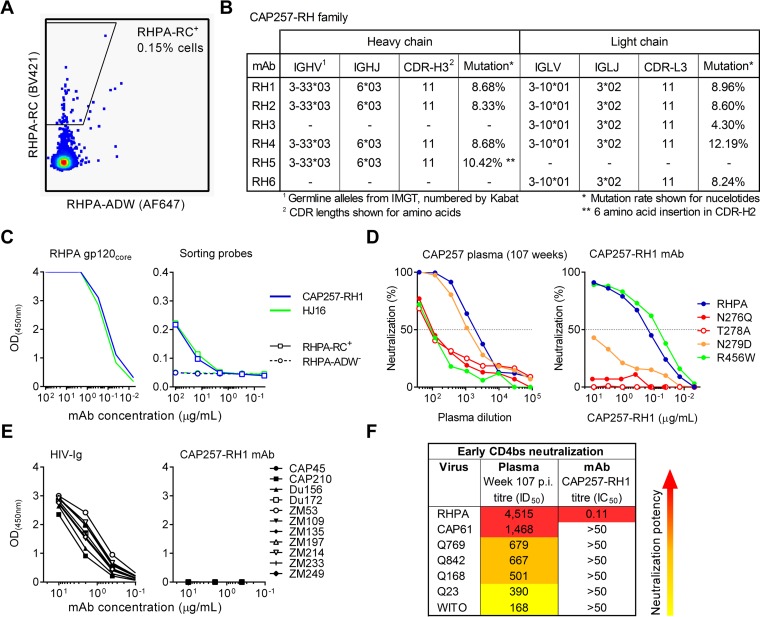
Isolation of an early N276 glycan-dependent CD4bs neutralizing antibody. (A) Sort plot showing the percentage of identified memory B cells that were able to bind the positive sorting antigen RHPA-RC (fluorescence plotted on the *y* axis) but not to the negative sorting antigen RHPA-ADW (fluorescence plotted on the *x* axis). (B) Properties of the CAP257-RH lineage members, defined by the International Immunogenetics database (IMGT) and numbered according to Kabat numbering. (C) Comparison of the binding of CAP257-RH1 or HJ16 to wild-type RHPA core gp120 (no symbols) as well as to the positive gp120 sorting antigen RHPA-RC or the negative gp120 sorting antigen RHPA-ADW by ELISAs. Absorbance is plotted on the *y* axis versus the antibody concentration on the *x* axis. (D) Neutralization of RHPA and select mutants with various levels of resistance to CAP257 CD4bs plasma antibodies by either donor CAP257 plasma at 107 weeks postinfection or the CAP257-RH1 monoclonal antibody isolated at the same time point. Percent neutralization is plotted on the *y* axis, and the dilution is plotted on the *x* axis. (E) ELISA binding of an anti-HIV immunoglobulin pool (positive control) or monoclonal antibody CAP257-RH1 to 11 clade C gp120 proteins. (F) Neutralization of seven heterologous viruses sensitive to early CAP257 CD4bs antibodies by either donor CAP257 plasma at 107 weeks postinfection (p.i.) or monoclonal antibody CAP257-RH1. Increasing potency is indicated by warmer colors.

A PBMC sample obtained at 107 weeks postinfection, when the CD4bs lineage was likely to be expanding, was selected for B-cell isolation. Single memory B cells were sorted as CD3^−^ CD14^−^ CD16^−^ IgD^−^ RHPA-ADW^−^ CD19^+^ RHPA-RC^+^ by flow cytometry (Fig. 2A). At least one antibody heavy or light chain could be recovered from 65% of the 94 sorted cells, and DNA sequencing revealed that RHPA-RC selected for three antibody lineages. The first of these lineages was a family of five IGHV4-59/61 heavy chains that were 16% to 18% mutated from the germ line and shared the same heavy chain family as CD4bs antibody CH103 (see Fig. S2A in the supplemental material). However, the paired light chains for this antibody family could not be recovered. Similarly, a family of six unpaired IGKV1-NL1 light chains that were between 5% and 16% mutated were also isolated but could not be functionally assessed without complementary heavy chain sequences (see Fig. S2B in the supplemental material). The third family was derived from IGHV3-33 heavy and IGLV3-10 light chains and is referred to here as the CAP257-RH lineage (Fig. 2B; see also Fig. S2C in the supplemental material). CAP257-RH heavy and light chain genes were 8 to 11% and 4 to 13% mutated, respectively, and had relatively normal CDR-H3 and CDR-L3 lengths of 11 amino acids each (Kabat numbering). One of these antibodies, CAP257-RH1, bound well to the wild-type RHPA gp120 core by ELISAs and bound weakly to the positive sorting antigen RHPA-RC (similarly to bNAb HJ16) but did not bind to the negative sorting antigen RHPA-ADW (Fig. 2C), confirming the specific selection of CAP257-RH lineage antibodies.

### CAP257-RH1 displays strain-specific heterologous neutralization and gp120 binding.

We have previously shown that escape from CAP257 CD4bs antibodies was due to the sequential appearance of three mutations at positions 279, 276/278, and 456. The N279D immunotype switch appeared first and preceded the acquisition of breadth. Mutations that deleted the glycosylation sequon at position N276 (e.g., T278A) appeared next, while the R456W substitution was the last major escape mutation (36). These CD4bs mutants were used to compare the neutralization of MAb CAP257-RH1 to a contemporaneous plasma sample from 107 weeks postinfection (Fig. 2D). The plasma samples obtained at 107 weeks postinfection neutralized RHPA at ID_50_ titers exceeding 1:1,000 and by this time had already adapted to tolerate the early D279 escape mutation but were still dependent on N276 and R456 for effective neutralization (Fig. 2D, left). CAP257-RH1 potently neutralized RHPA with an IC_50_ of 0.11 μg/ml (Fig. 2D, right). This neutralization was completely abrogated by N276Q and T278A mutations that removed the N276 glycan, confirming that CAP257-RH1 was a component of the CAP257 CD4bs plasma neutralizing response (Fig. 2D). However, unlike CAP257 plasma, CAP257-RH1 neutralization was substantially reduced (100-fold) by the early N279D mutation and unaffected by the late R456W change. These data suggest that CAP257-RH1 appeared very early in the CAP257 CD4bs neutralizing response, before the emergence of CD4bs-specific neutralization breadth.

The cross-reactivity and breadth of MAb CAP257-RH1 were further examined by ELISAs and neutralization assays, respectively (Fig. 2E and F). CAP257-RH1 showed binding to RHPA gp120 but was unable to bind any of the 11 heterologous clade C gp120 monomers tested, all of which were bound by a pooled HIV-positive (HIV^+^) immunoglobulin control (HIV-Ig) (Fig. 2E). Similarly, CAP257-RH1 displayed strain-specific heterologous neutralization of only RHPA among the 196-virus multiclade panel tested, including other viruses neutralized by early CAP257 CD4bs neutralizing antibodies (Fig. 2F; see also Table S1 in the supplemental material). CAP257-RH1 did not display incomplete neutralization maxima, as seen with a number of recently isolated glycan-dependent HIV-1 bNAbs (45).

### Structural classification of CAP257-RH1 as a CDR-H3-dominated class CD4bs antibody.

**FIG 3:**
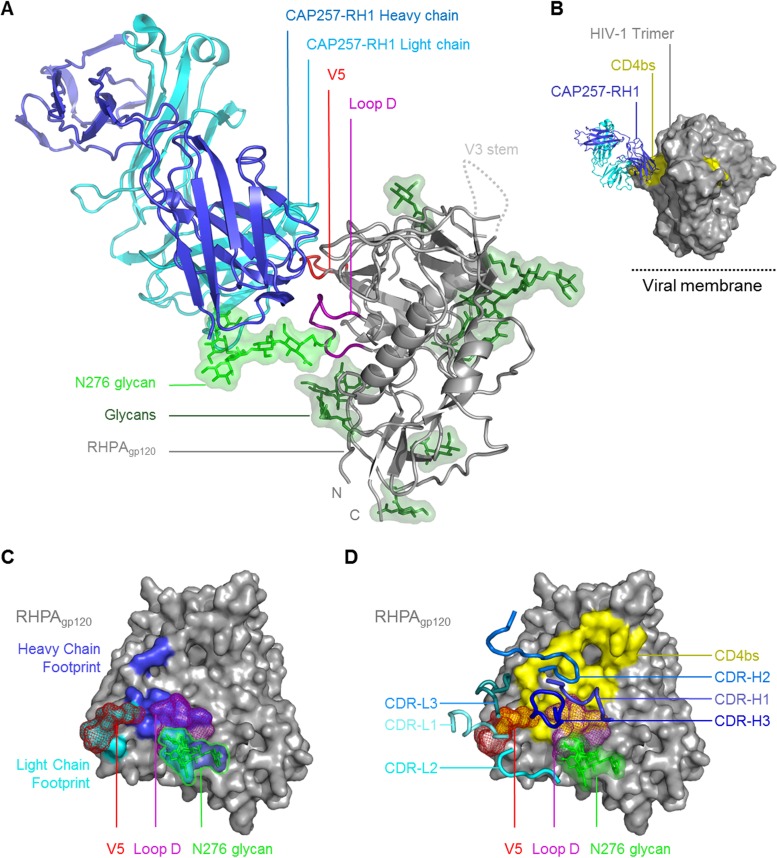
Crystal structure of CAP257-RH1 bound to the N276 glycan in RHPA gp120. (A) Overview of the CAP257-RH1 Fab and RHPA core gp120 cocrystal structure shown in a cartoon view. The truncated N/C termini as well as the V3 loop stem are labeled. Glycans are shown in stick view with green semitransparent surfaces. The N276 glycan is indicated by its lighter shade. (B) Surface view of the HIV-1 Env trimer (PDB accession no. 4TVP) with monoclonal antibody CAP257-RH1 docked into its epitope (by superimposition). The location of the viral membrane is indicated. (C) Surface view of RHPA gp120. Loop D and V5 are indicated, and the N276 glycan (shown in stick-and-surface view) is outlined. (D) Surface view of RHPA gp120 with the CD4 binding footprint. Loop D, V5, and the N276 glycan are indicated as described above for panel C. The CAP257-RH1 CDRs are shown as thick blue cartoon loops. For clarity, the protein chain is indicated in subscript (heavy chain, light chain, or gp120).

**FIG 4:**
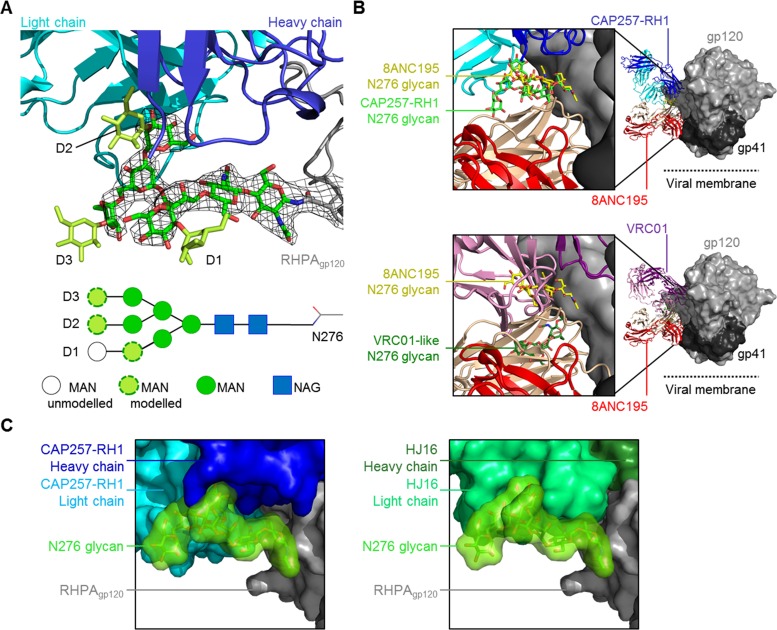
Analysis of the N276 glycan bound by neutralizing antibodies. (A) CAP257-RH1 shown in cartoon view, as described in the legend of Fig. 3, interacting with the N276 glycan (green sticks). The 2*F*_o_ − *F*_c_ electron density map (contoured at 1 σ) of the N276 glycan is shown with black mesh. Additional mannose moieties added to the glycan termini are shown in lime green, and the glycan arms are labeled. A schematic of the glycan is also shown at the bottom, and sugars with visible density or those modeled onto the structure are labeled. (B) Comparison of the orientations of the N276 glycan bound by 8ANC195 (with Fab heavy and light chains in peach and red, respectively), CAP257-RH1 (with Fab heavy and light chains in blue and cyan, respectively), or VRC01-like antibodies (with Fab heavy and light chains in purple and pink, respectively). The Fab-bound glycan complexes (PDB accession no. 4P9H and 4JKP) were superimposed onto the HIV-1 Env trimer structure reported under PDB accession no. 4TVP and are shown in light gray (gp120) and dark gray (gp41) surface views. (C, left) Surface view of the N276 glycan bound by CAP257-RH1 (colored as for panel A). (Right) HJ16 cocrystal structure (PDB accession no. 4YE4) superimposed onto CAP257-RH1-bound gp120 showing compatibility with this N276 glycan orientation.

A cocrystal structure of the CAP257-RH1 Fab region bound to RHPA core gp120 was determined to 3.2-Å resolution (Table 1 and Fig. 3A). The structure included an (*N*-acetylglucosamine)_2_(mannose)_5_ (NAG_2_MAN_5_) glycan at position N276 bound in the paratope (Fig. 3A, light green) and confirmed the CD4bs as the target for CAP257-RH1 neutralization. The crystallization conditions included Endo H to facilitate slow, in-drop deglycosylation of gp120, since preincubation with Endo H disrupted the Fab-gp120 complexes; however, the enzyme was present at a very low concentration and did not contribute to the asymmetric unit. The CAP257-RH1 angle of approach was compatible with Env oligomerization and did not clash with the V1V2 domain (Fig. 3B), as was seen previously for other narrowly neutralizing CD4bs antibodies (46, 47). The interface between CAP257-RH1 and RHPA gp120 buries a total surface area of ∼1,184 Å^2^, to which the heavy chain contributes ∼593 Å^2^ and the light chain contributes ∼594 Å^2^ (Fig. 3C; see also Tables S2 to S4 in the supplemental material). Unlike other CD4bs antibodies, CAP257-RH1 does not extend its CDRs deep into the cavity bound by CD4 but instead interacts with more peripheral regions of the CD4bs, including loop D, V5, and the CD4 binding loop (Fig. 3C and D; see also Fig. S3 in the supplemental material). Accordingly, protein-protein interactions with gp120 account for ∼748 Å^2^ of the total buried surface area. More than one-third of the total interface (∼436 Å^2^) involves interactions with the N276 glycan, which fits snugly at the heavy chain-light chain interface, where each chain contributes ∼201 Å^2^ and ∼235 Å^2^ of bound surface area, respectively. This area is similar to the 300 Å^2^ to 400 Å^2^ buried by N276 in the interfaces of some VRC01 relatives, which are not glycan dependent, but is only half as large as the interface with N276 made by 8ANC195 (48–50). The gp120 used for these crystallization experiments was expressed in HEK293S GntI^−/−^ cells to facilitate deglycosylation at pH 7. As a result, the cocrystal structure includes a low-mannose glycan bound in the CAP257-RH1 paratope. Previously, the N276 glycan on monomeric gp120 was characterized as being mostly complex in composition (51–53), but a more recent analysis of native prefusion trimers suggests that this glycan is mostly oligomannose in composition and varies from MAN_5_ to MAN_8_ (54). Additional mannose residues could be modeled at the termini of each glycan arm in the CAP257-RH1-bound structure, suggesting that this antibody's binding was compatible with both NAG_2_MAN_5_ low-mannose and NAG_2_MAN_8/9_ high-mannose glycans at position N276 (Fig. 4A). In both the CAP257-RH1- and 8ANC195-bound structures, the N276 glycan adopts a similar orientation that differs substantially from the reoriented glycan conformation bound by VRC01-like antibodies (Fig. 4B). Similarly, the CAP257-RH1-bound orientation of the N276 glycan (Fig. 4C, left) was compatible with HJ16-bound gp120 (by superimposing the gp120 molecules from both structures) and packed closely against the HJ16 light chain in the model (Fig. 4C, right), which is indicative of a common N276 glycan conformation bound by these neutralizing antibodies. Thus, this structure of the N276 glycan may represent a commonly occupied orientation of the N276 glycan bound by bNAbs.

**TABLE 1:**
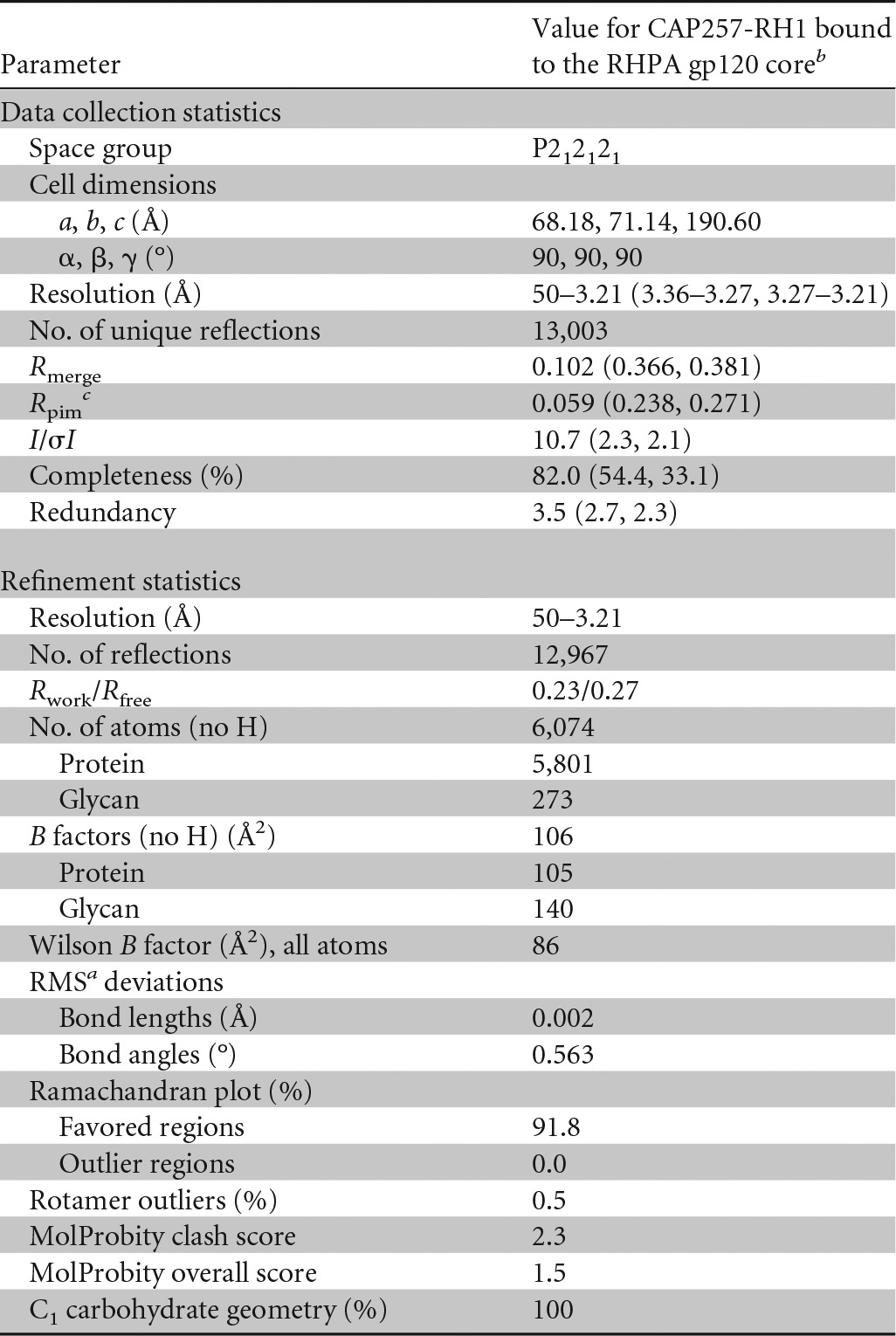
X-ray crystallographic data and refinement statistics (molecular replacement) for the antigen binding fragment of CAP257-RH1 in complex with the HIV-1 RHPA gp120 core

^a^ RMS, root mean square.

^b^ Values in parentheses are for the highest-resolution shells.

^c^ Precision-indicating merging *R* value.

**FIG 5:**
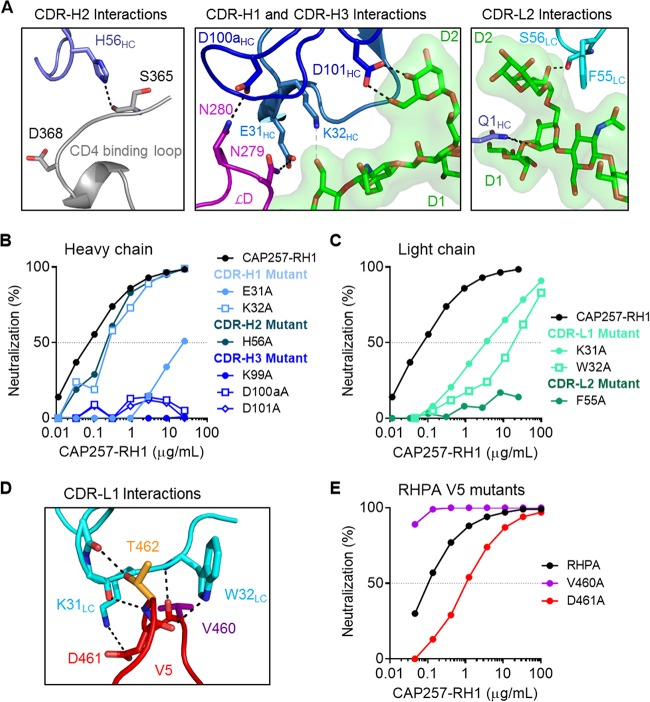
Atomic details of the CAP257-RH1 interaction with RHPA core gp120. (A) Polar contacts between CAP257-RH1 and the CD4bs/N276 glycan. The CAP257-RH1 CDRs are shown in various shades of blue, while the CD4b binding loop, loop D, and the N276 glycan are shown in gray, purple, and green, respectively. Potential hydrogen bonds are shown as black dotted lines. (B) Paratope mapping of the CAP257-RH1 heavy chain by alanine scanning mutagenesis. Mutations are grouped by CDR loop using different shades of blue. Percent inhibition is plotted on the *y* axis, and the antibody concentration is plotted on the *x* axis. (C) Paratope mapping of the CAP257-RH1 light chain, as described above for panel B. (D) Atomic details of the interaction between CAP257-RH1 CDR-L1 (cyan) and the V5 loop of RHPA (red). Polar interactions are indicated as described above for panel A. (E) Neutralization of various RHPA alanine mutants, plotted as described above for panel B.

The CAP257-RH1 paratope is centered on its 11-amino-acid-long CDR-H3, which accounts for ∼53% of the heavy chain interface (Fig. 3D). The remaining heavy chain CDR loops were positioned to interact with the conserved CD4bs, while the light chain CDRs were more distal, in close proximity to V5. In concordance with data from plasma mapping, CAP257-RH1 does not interact productively with D368_gp120_ despite having a CDR-H2 that extended toward the CD4 binding loop (similarly to VRC01), where H56_HC_ forms a hydrogen bond with the S365_gp120_ backbone carbonyl oxygen (Fig. 5A, left). The CDR-H1 and CDR-H3 loops are both in close proximity to gp120 loop D, where E31_HC_ and D100a_HC_ make hydrogen bonds with N279_gp120_ and N280_gp120_, respectively (Fig. 5A, middle). The interaction between E31_HC_ and N279_gp120_ explains the antibody's preference for an N279_gp120_ immunotype, as a negatively charged aspartic acid immunotype at position 279_gp120_ would clash electrostatically with the glutamic acid in CDR-H1. CDR-H3 also makes contact with the peptide backbone of loop D and V5 through K99_HC_ and with the D2 arm of the N276 glycan through D101_HC_, while K32_HC_ in CDR-H1 is positioned to make weak contact with the first NAG moiety. CAP257-RH1 also contacts the D1 arm of the N276 glycan via its heavy chain N-terminal residue (Fig. 5A, right). In addition to these heavy chain interactions, CDR-L2 of the light chain also contributes to N276 glycan interactions by inserting F55_LC_ between the second NAG moiety and the D2 arm of the glycan as well as a hydrogen bond with the backbone carbonyl of S56_LC_ (Fig. 5A, right).

To evaluate the relative contributions of each of the contacts identified above, alanine scanning mutants of the CAP257-RH1 paratope were made in the heavy chain (Fig. 5B) or light chain (Fig. 5C) and assessed for their ability to neutralize RHPA. Heavy chain mutations at K32_HC_ in CDR-H1 and at H56_HC_ in CDR-H2 marginally affected CAP257-RH1 neutralization, while the E31A_HC_ mutation had a much more substantial effect (Fig. 5B). The reduction in the potency of the E31A_HC_ mutant against RHPA was equivalent to that of the wild-type antibody against the N279D_gp120_ mutant virus, consistent with the observed interaction between these two residues (compare Fig. 2D and 5B). In contrast to the CDR-H1/H2 contributions, all of the CDR-H3 mutants failed to neutralize RHPA, highlighting the important role of this region in mediating neutralization. These data support the classification of CAP257-RH1 as having a CDR-H3-dominated paratope despite having a CDR-H3 loop length that is much shorter than those of other HIV-1 CD4bs antibodies of this class.

The CDR-L2 F55A_LC_ mutant, which disrupts interactions with the N276 glycan, was also unable to neutralize RHPA (Fig. 5C). Two additional light chain contacts in CDR-L1 at positions K31_LC_ and W32_LC_ also had a substantial contribution to CAP257-RH1 neutralization, but alanine mutations at these sites did not completely abrogate the antibody's activity (Fig. 5C). While interactions with the conserved CD4bs (loop D and the CD4 binding loop) are mediated exclusively by the CAP257-RH1 heavy chain, the light chain is positioned close to V5, with its CDR-L1 binding perpendicularly over the V5 loop apex (Fig. 5D). Light chain interactions with V5 account for ∼259 Å^2^ of buried surface area and include extensive hydrogen bonding with the V5 peptide backbone and notable van der Waals contributions by the W32_LC_ side chain, which inserts behind V5 to contact the backbone carbonyl of D461_gp120_. This displaces the D461_gp120_ side chain into the CAP257-RH1 paratope, where it interacts with K31_LC_ (Fig. 5D). However, mutation of D461_gp120_ to alanine in the RHPA V5 loop affected the IC_50_ but was not critical for neutralization, while mutation of V460_gp120_ actually enhanced CAP257-RH1 neutralization (Fig. 5E). Taken together, both heavy and light chain interactions with the N276 glycan (D101_HC_ and F55_LC_ but not K32_HC_), loop D (E31_HC_ and D100a_HC_), or the base of V5 (K99_HC_) were critical for CAP257-RH1 neutralization, while specific contacts with the hypervariable V5 loop apex (K31_LC_ and W32_LC_) were not essential for neutralization and thus not the primary determinant of CAP257-RH1 strain specificity.

### CAP257-RH1 neutralization is incompatible with glycosylated V5 loops.

**FIG 6:**
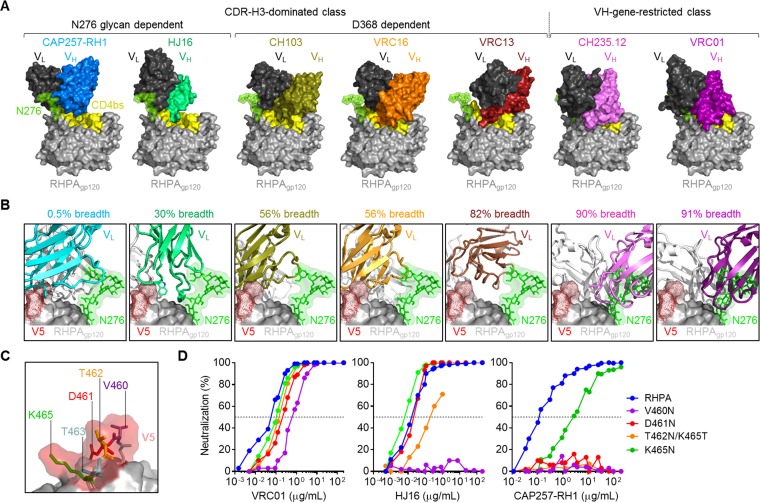
CAP257-RH1 neutralization is constrained by V5 glycosylation. (A) Comparison of the relative levels of penetration into the recessed CD4bs for various CD4bs neutralizing antibodies (crystal structures determined previously) superimposed onto the RHPA crystal structure, shown in surface view. The antibodies are grouped as CDR-H3 dominated or VH gene restricted and ordered according to increasing neutralization breadth. N276 glycan-dependent antibodies are indicated. For all antibodies, the light chains are shown in black, while the heavy chains are shown in various colors. (B) Comparison of the binding angles for the CD4bs antibodies shown in panel A relative to RHPA V5, with the CAP257-RH1-bound N276 glycan shown as sticks with a semitransparent surface. All antibodies are shown in cartoon view, with the heavy chains in white and the light chains colored according to neutralization breadth, as described above for panel A. Previously reported neutralization breadth estimates are shown. (C) Zoomed view of the glycan-free RHPA V5 loop shown as sticks with a transparent surface, with amino acids 460 to 464 being labeled. (D) Neutralization of RHPA and glycosylated V5 mutants by VRC01, HJ16, and CAP257-RH1. Percent inhibition is plotted on the *y* axis, and the antibody concentration is plotted on the *x* axis.

To better understand the strain specificity of CAP257-RH1, the cocrystal structures of other CD4bs antibodies were superimposed onto the cocrystal structure of CAP257-RH1 bound to RHPA gp120 (Fig. 6). Both N276 glycan-dependent antibodies CAP257-RH1 and HJ16 infiltrated less substantially into the CD4bs than the other CD4bs neutralizing antibodies (Fig. 6A). The VH gene-restricted bNAbs VRC01 and CH235.12 were predicted to clash significantly with the CAP257-RH1-bound orientation of the glycan at position N276, but the placement of this glycan was compatible with all of the antibodies of the CDR-H3-dominated class. These data are consistent with previously reported observations that VH gene-restricted bNAbs bind better to N276 glycan-deficient Env (31, 32, 55). Another striking difference between the CD4bs antibodies was their binding orientations relative to V5. While VH gene-restricted CD4bs antibodies completely avoided the V5 loop apex, the binding of CDR-H3-dominated antibodies often placed their light chains in close proximity to V5 (Fig. 6B). Overall, less penetration into the recessed CD4bs and greater overlap of the V5 loop apex correlated with reduced neutralization breadth. This was particularly prominent for N276 glycan-dependent CD4bs antibodies HJ16 and CAP257-RH1, both of which increased their interacting surfaces with V5 through a bulky tryptophan residue from the CDR-L3 or CDR-L1 loop, respectively (shown in Fig. 6B). The extent of this overlap suggested that the binding angles for these two antibodies were less tolerant of longer or more glycosylated V5 loops than other CD4bs antibodies such as VRC01.

The RHPA V5 loop is relatively short (5 amino acids between positions 460 and 464) and unusually glycan-free (Fig. 6C). Indeed, glycan-free V5 loops make up only 6% of the ∼5,000 HIV-1 group M Env sequences in the LANL database. To test whether this lack of glycan contributed to CAP257-RH1 strain specificity, V5-glycosylated sequence variants of RHPA were made and tested for their sensitivity to CAP257-RH1, HJ16, and VRC01 (Fig. 6D). All these V5 mutants were more resistant to the three CD4bs antibodies tested, although the effect varied depending on the location of the introduced glycosylation sequon. All the mutants were marginally less sensitive to neutralization by VRC01, and this effect was more pronounced for V5 N-terminal glycan substitutions. In contrast, HJ16 neutralization was knocked out by a glycan at position 460_gp120_ and substantially affected by a glycan site at position 462_gp120_, while CAP257-RH1 neutralization was completely abrogated by the introduction of glycosylation sequons at positions 460_gp120_, 461_gp120_, and 462_gp120_ and significantly affected by a glycan at position 464_gp120_. A glycosylation sequon at position 463_gp120_ could not be tested because mutation of E465_gp120_ resulted in nonfunctional virions. These data are consistent with the level of V5 overlap displayed by each of these antibodies and support the hypothesis that CAP257-RH1 neutralization breadth is substantially limited by V5 glycosylation.

### A minority population of CAP257 autologous viruses lacks glycans in V5.

**FIG 7:**
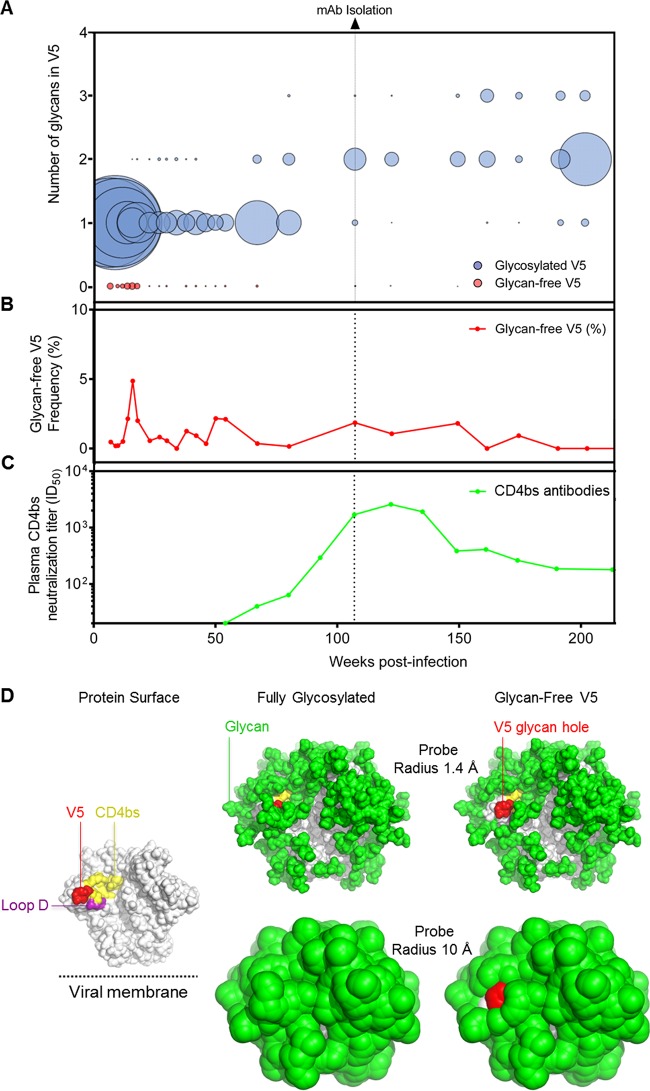
Rare glycan-free V5 sequences from CAP257 reveal a hole in the glycan shield. (A) Deep sequencing of autologous V5 loop sequences in donor CAP257, with the number of glycans on the *y* axis and weeks postinfection on the *x* axis. The relative frequencies of the number of glycans in V5 at a particular time point are represented by the size of the spheres, where larger spheres indicate a greater relative number of sequences at that particular time point that have the indicated number of glycosylation sequons in V5. The dotted vertical line indicates the time point at which CAP257-RH1 was isolated. (B) Relative number of glycan-free autologous V5 loops plotted as a percentage of the total sequences (*y* axis) over time (*x* axis). (C) Neutralization titers (plotted on the *y* axis) of the second wave of broadly neutralizing antibodies from donor CAP257 plasma (as described in reference 36), which target the CD4bs, over time (*x* axis). (D, left) The HIV-1 Env trimer is shown in a solvent-accessible surface representation (gray). The approximate location of the viral membrane is indicated. (Right) The glycan shield is modeled and displayed as the solvent-accessible surface of NAG_2_MAN_9_ glycan coverage based on two probe sizes of 1.4 Å (solvent radius) (top) and 10.0 Å (the estimated steric footprint of an antibody-combining region) (bottom), using the structure reported under PDB accession no. 4TVP with an additional glycosylation site modeled at residue 241. The solvent-accessible protein surface of V5 comprises a largely exposed region upon the deletion of the commonly glycosylated site at residue 462 (right).

Donor CAP257 was infected with a clade C tier 2 virus, which, from the earliest autologous V5 loop consensus (7 weeks postinfection), was glycosylated at position 461_gp120_ and consequently resistant to CAP257-RH1 neutralization (see Tables S1 and S5 in the supplemental material). To identify potential autologous glycan-free V5 loops, a panel of ∼150 CAP257 Env sequences isolated by single-genome amplification (SGA) at 16 time points throughout infection of donor CAP257 were examined, but all the sequences were glycosylated, making it unclear which virus stimulated the CAP257-RH lineage (see Fig. S4 in the supplemental material). The autologous CAP257 viral sequences were further probed by deep sequencing of the V3-to-V5 region of Env from 25 time points between 7 weeks (study enrollment) and 202 weeks postinfection, resulting in 19,761 consensus V5 sequences (Fig. 7A). The CAP257-RH lineage was isolated at 107 weeks postinfection, where the major viral population had two glycans per V5 loop. However, at time points between 7 weeks and 67 weeks postinfection, a minor population of CAP257 viruses was detected, with completely glycan-free V5 loops that were dominated by a D461 immunotype (Fig. 7A, red spheres). These sequences peaked in frequency (at 4.9% of total sequences) at 16 weeks postinfection (Fig. 7B), when the viral load was ∼32,000 copies/ml (equating to ∼1,568 RNA copies encoding glycan-free V5 Env sequences per ml of blood), but occurred less frequently after the detection of CAP257 CD4bs neutralizing plasma antibodies at 54 weeks postinfection (Fig. 7C). A population of glycan-free V5 loops was consistently detectable at very low levels (<1% of total sequences) thereafter, alongside the evolving CAP257 CD4bs neutralizing antibody response, but appeared to be spontaneously generated and was unrelated to the initial glycan-free V5 loops detected prior to the development of CD4bs antibodies.

To evaluate the potential effect of these glycan-free V5 loops on the Env glycan shield, we modeled the surface coverage of NAG_2_MAN_9_ glycans on the Env trimer. Accessibility to the underlying protein surface was evaluated by using probe radii of either 1.4 Å (the radius of a water molecule) or 10 Å (the approximate radius of a single Fab domain) (Fig. 7D). This analysis revealed an unprotected region of the Env protein surface, showing how a glycan hole in V5 exposes epitopes for the induction of autologous neutralizing antibodies such as CAP257-RH1.

## DISCUSSION

The CD4bs on the HIV-1 envelope is the most extensively studied site of vulnerability to antibody neutralization, but less is known about how these antibodies arise in infection or how they might be induced by vaccination. Here, we isolated and characterized a CD4bs antibody called CAP257-RH1, which recognizes an N276 glycan-dependent epitope targeted by early members of the CD4bs plasma bNAb response that developed in donor CAP257. A cocrystal structure that included the N276 glycan revealed extensive contacts between the CAP257-RH1 heavy chain and conserved elements of the CD4bs. Narrow neutralization breadth was attributed to a light chain binding angle that was incompatible with glycosylated V5 loops present in almost all HIV-1 strains. Rare autologous sequences from donor CAP257 early in infection were found to have glycan-free V5 loops that may have been responsible for engaging the naive B cell that produced this CD4bs antibody lineage. Thus, vaccine immunogens that lack glycans in V5 may provide better exposure for the epitopes targeted by N276 glycan-dependent CD4bs antibodies.

Although the CAP257-RH1 monoclonal antibody is strain specific for the heterologous virus RHPA, it targets the same N276 glycan-dependent CD4bs epitope as CAP257 plasma bNAbs. In addition, CAP257-RH1 was very sensitive to the N279D mutation that in plasma affected only the earliest CAP257 CD4bs neutralizing antibodies and coincided with the emergence of the later broadly neutralizing CD4bs response. Thus, CAP257-RH1 likely represents an early member of the neutralizing response to the CD4bs in this individual. Three studies detailing the ontogeny of bNAbs showed that they arose from strain-specific precursors (17, 18, 20). While strain-specific neutralizing antibodies have long been known to target the hypervariable regions of Env (2, 3, 5–8), it has become apparent that the epitopes for strain-specific antibodies may also substantially overlap the epitopes for bNAbs (56, 57). For instance, 2909 is strain specific for the heterologous virus SF162 and targets an epitope in V2 (58), but the isolation of PG9 and PG16 showed that this epitope was also vulnerable to bNAbs (59). The limited neutralization breadth of 2909 could be explained by the requirement for a lysine at position 160 (normally glycosylated) creating a hole in the Env glycan shield (57). CAP257-RH1 specificity was similarly constrained by the requirement for a glycan-free V5 loop and a slight preference for the D461 immunotype. However, CAP257-RH1 still neutralized V5 alanine mutants of RHPA and could conceivably mature to recognize more diverse V5 loop sequences. Alternatively, these antibodies may have helped drive viral diversity in the CD4bs, in turn promoting the development of plasma neutralization breadth, similar to the cooperative evolution of the CH103 and CH235 lineages in donor CH505 (21). It is not known whether the CAP257-RH lineage also went on to develop broadly neutralizing activity, and the isolation of CD4bs bNAbs from donor CAP257 would help to address this question.

CAP257-RH1 together with HJ16 and 179NC75 form a subclass of CDR-H3-dominated CD4bs antibodies (26, 28) that are critically dependent on the N276 glycan but not D368 (28, 36, 60). CAP257-RH1 binds the N276 glycan in a recess created by the antibody heavy chain-light chain interface and recognizes the glycan with an orientation similar to that of bNAb 8ANC195 that targets the gp120-gp41 interface (49, 61). This glycan orientation could also be accommodated by glycan-dependent CD4bs antibody HJ16 in our modeling analysis but not by the VH gene-restricted antibodies, which clash sterically with N276, consistent with previously reported crystal structures showing the relocation of this glycan by VRC01 and related antibodies (48, 50). The degree of similarity in N276 glycan recognition is comparable to that of the N332 glycan supersite, which is oriented in the same conformation by antibodies with very different epitopes and angles of approach (62). Together with more recent insights into the structure of the glycan shield (50, 54), these data help to define the role of N276 in bNAb development. HJ16 and other CDR-H3-dominated class bNAbs increase interactions with the CD4 binding pocket through long CDR-H3 loops. CAP257-RH1 binds to the periphery of the CD4bs but does not extend deep into the conformationally occluded CD4 binding pocket, and its deviation from the previously described CD4bs of vulnerability results in a narrow neutralization breadth.

CAP257-RH1 was unable to neutralize viruses with glycosylated V5 loops, including the CAP257 transmitted/founder virus. We speculate that the CAP257-RH lineage was elicited by low-frequency autologous variants that had glycan-free V5 loops. These viruses never came to dominate the viral population, possibly due to selection pressures imposed by the CAP257-RH lineage. Alternatively, it is also possible that rare glycan-skipping events in V5 (where glycosylation sequons are missed by oligosaccharyltransferase in the Golgi apparatus) stimulated the lineage. Interestingly, in another individual (donor CAP256) who developed broad and potent V2-reactive antibodies from a strain-specific precursor (17, 63), the bNAb-initiating Env was also found to be relatively rare compared to the dominantly circulating viral variant (64). Similarly to the CAP257-RH1 epitope, several other studies have also identified strain-specific neutralizing antibody targets created by unusual holes in the glycan shield (22, 58, 65, 66). These targets include vaccine-induced antibodies (67, 68), such as the strain-specific macaque antibody DH427, which targets a large glycan hole in both V5 and loop E, allowing an angle of approach that does not include the CD4bs, and therefore is unlikely to mature toward neutralization breadth. In donor 45, from which VRC01 was isolated, proviral sequences with a rare hole in the glycan shield at position N276 were identified (69, 70), which may represent rare bNAb-initiating Envs that set off the VRC01-like lineages in this donor. Thus, glycan holes may elicit highly strain-specific responses that are unable to further mature toward neutralization breadth but may also represent key features of HIV-1 immunogens that engage the appropriate precursors of HIV-1 bNAbs. While these holes may recruit the appropriate naive B cells, driving the process toward breadth will likely require the addition of fully glycosylated native trimeric immunogens that select for mutations that are able to accommodate the Env glycan shield (34, 35).

Immunogens designed to activate VRC01 class VH gene-restricted CD4bs bNAb precursors have so far shown promising results in animal studies. These antigens not only lack the N276 glycan but also have been modified to bind with higher affinity to the VH1-2 germ line gene (31, 32). Germ line gene knock-in mice immunized with these constructs produced antibodies with neutralization-compatible mouse light chains (34, 35), and germ line-targeting antigens preferentially select for VH gene-restricted precursors in healthy individuals (71). In addition to N276 mutants, Env immunogens that also lack glycans in V5 bind even better to the VH gene-restricted CD4bs bNAb precursors (32). In this study, we show that viruses lacking glycans in V5 are also considerably better neutralized by N276 glycan-dependent antibodies. This subclass of CD4bs antibodies would not be elicited by the N276-deficient Env immunogens designed to elicit VH gene-restricted antibodies. Based on the CAP257-RH1 cocrystal structure, glycan-free V5 may have been expressly required for activating the CAP257-RH lineage, and immunogens incorporating this feature may also improve binding to the precursors of other N276 glycan-dependent antibodies.

Altogether, these data provide insights into the N276 glycan as a target for HIV-1 neutralizing CD4bs antibodies and the types of immunogens that might be required to activate their naive B-cell precursors. Further isolation and characterization of N276 glycan-dependent bNAbs will be important for understanding how CD4bs immunogens might better capture the entire repertoire of bNAb precursors by immunization. Future studies will aim at assessing the role of glycan holes in priming B-cell responses that might mature to acquire broadly neutralizing activity.

## Supplementary Material

Supplemental material
