# Supplementary material for: Structure of an N276-Dependent HIV-1 Neutralizing Antibody Targeting a Rare V5 Glycan Hole Adjacent to the CD4 Binding Site
Source: J Virol. 2016 Oct 28;90(22):10220–35. doi: 10.1128/JVI.01357-16 (PMC5105658; doi:10.1128/JVI.01357-16)
Supplement: Supplemental material [file supp_90_22_10220__index.html]

Structure of an N276-Dependent HIV-1 Neutralizing Antibody Targeting a Rare V5 Glycan Hole Adjacent to the CD4 Binding Site — Supplemental material 

# Structure of an N276-Dependent HIV-1 Neutralizing Antibody Targeting a Rare V5 Glycan Hole Adjacent to the CD4 Binding Site

## Supplemental material

- Supplemental file 1 -

  Fig. S1 (Design of a resurfaced antigen based on RSC3 to isolate early CD4bs antibodies from donor CAP257.)

  Fig. S2 (Potentially related immunoglobulin clusters isolated from donor CAP257 at 107 weeks postinfection.)

  Fig. S3 (Residue-by-residue contacts between RHPA gp120 and CAP257-RH1.)

  Fig. S4 (Autologous CAP257 V5 sequences.)

  Table S1 (Heterologous neutralization mediated by CAP257-RH1.)

  Table S2 (CAP257-RH1-interacting residues.)

  Table S3 (RHPA gp120-interacting residues.)

  Table S4 (List of hydrogen bonds and salt bridges.)

  Table S5 (Frequency of glycosylation at position N276 and in the V5 loop of a multiclade 196-virus panel.)

  PDF, 2.9M
