# Supplementary material for: Structure of an N276-Dependent HIV-1 Neutralizing Antibody Targeting a Rare V5 Glycan Hole Adjacent to the CD4 Binding Site
Source: J Virol. 2016 Oct 28;90(22):10220–35. doi: 10.1128/JVI.01357-16 (PMC5105658; doi:10.1128/JVI.01357-16)
Supplement: Supplemental material [file JVI.01357-16_zjv999182096so1.pdf]

Supplementary Figure 1: Design of a resurfaced antigen based on RSC3 to isolate early CD4bs antibodies from donor CAP257

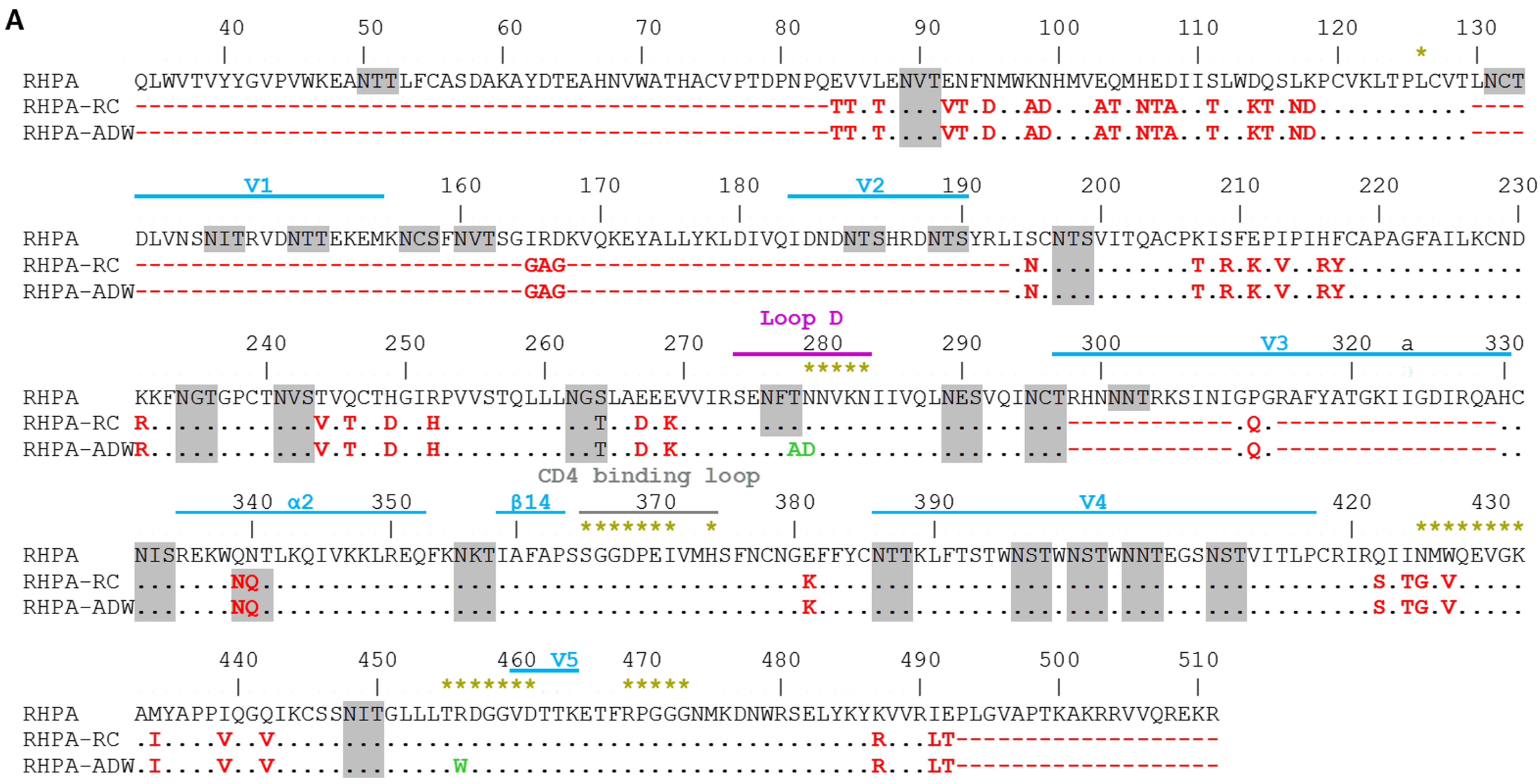

A) A sequence alignment of RHPA gp120, and the two newly designed sorting antigens RHPA-RC and RHPA-ADW. Truncations or resurfacing mutations are coloured red, potential N-linked glycosylation sequons are shaded grey, the hypervariable regions, loop D, and the CD4 binding loop are highlighted cyan, purple, and light grey respectively, and the CD4bs is indicated with yellow asterisks. The CAP257 escape mutations incorporated into the negative sorting antigen RHPA-ADW are coloured green.

Supplementary Figure 2: Potentially related immunoglobulin clusters isolated from donor CAP257 at 107 weeks post-infection

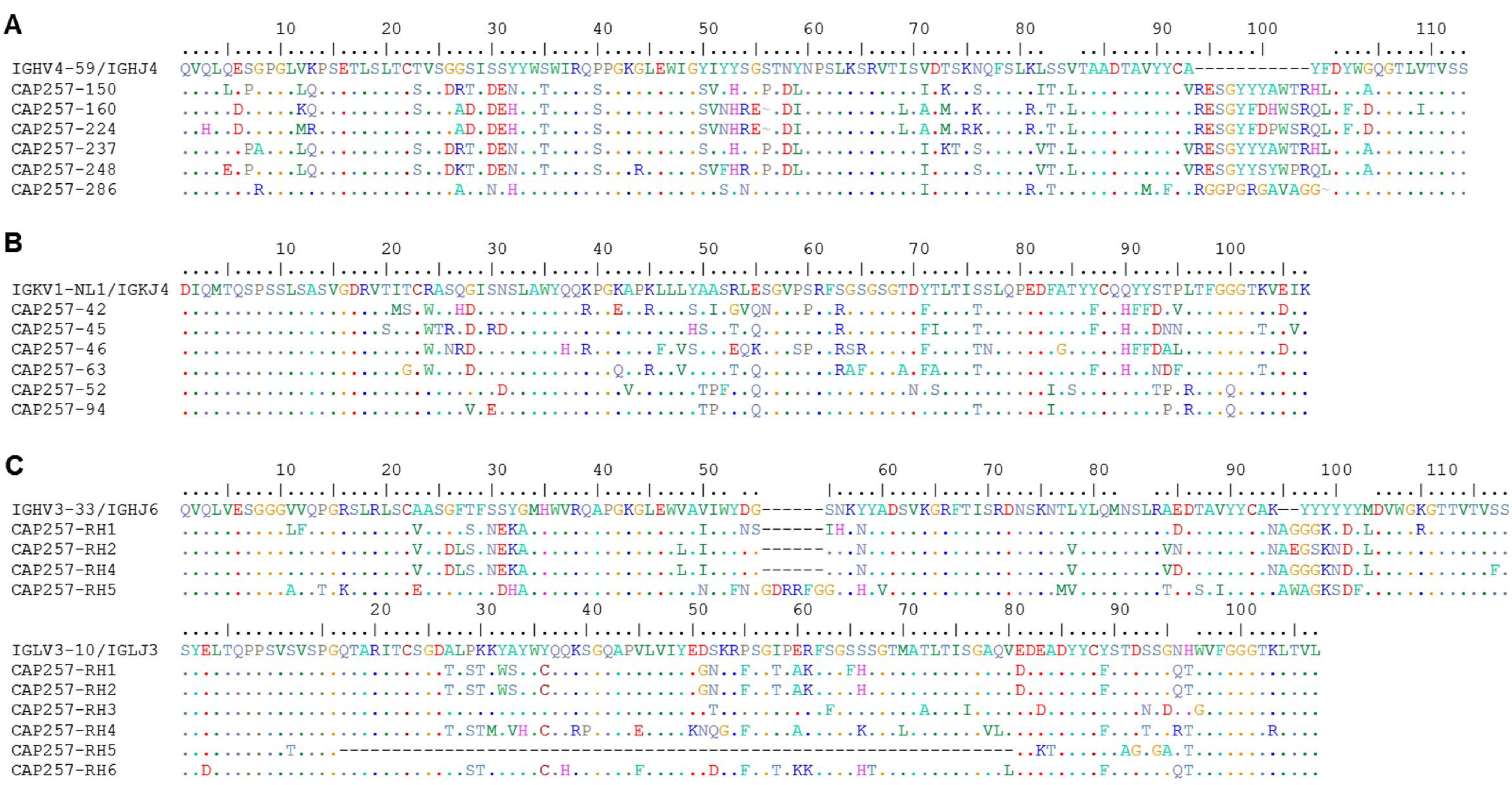

Sequence alignments of the three potentially related immunoglobulin clusters isolated as RHPA-RC+, RHPA-ADW- by flow cytometry from CAP257 PBMC memory B cells, numbered according to Kabat. A) Heavy chain alignment of the IGHV4-59 genes. B) Kappa chain alignment of the IGKV1-NL1 genes. C) Heavy and light chain alignments of the IGHV3-33 and IGLV3-10 genes.

Supplementary Figure 3: Residue-by-residue contacts between RHPA gp120 and CAP257-RH1

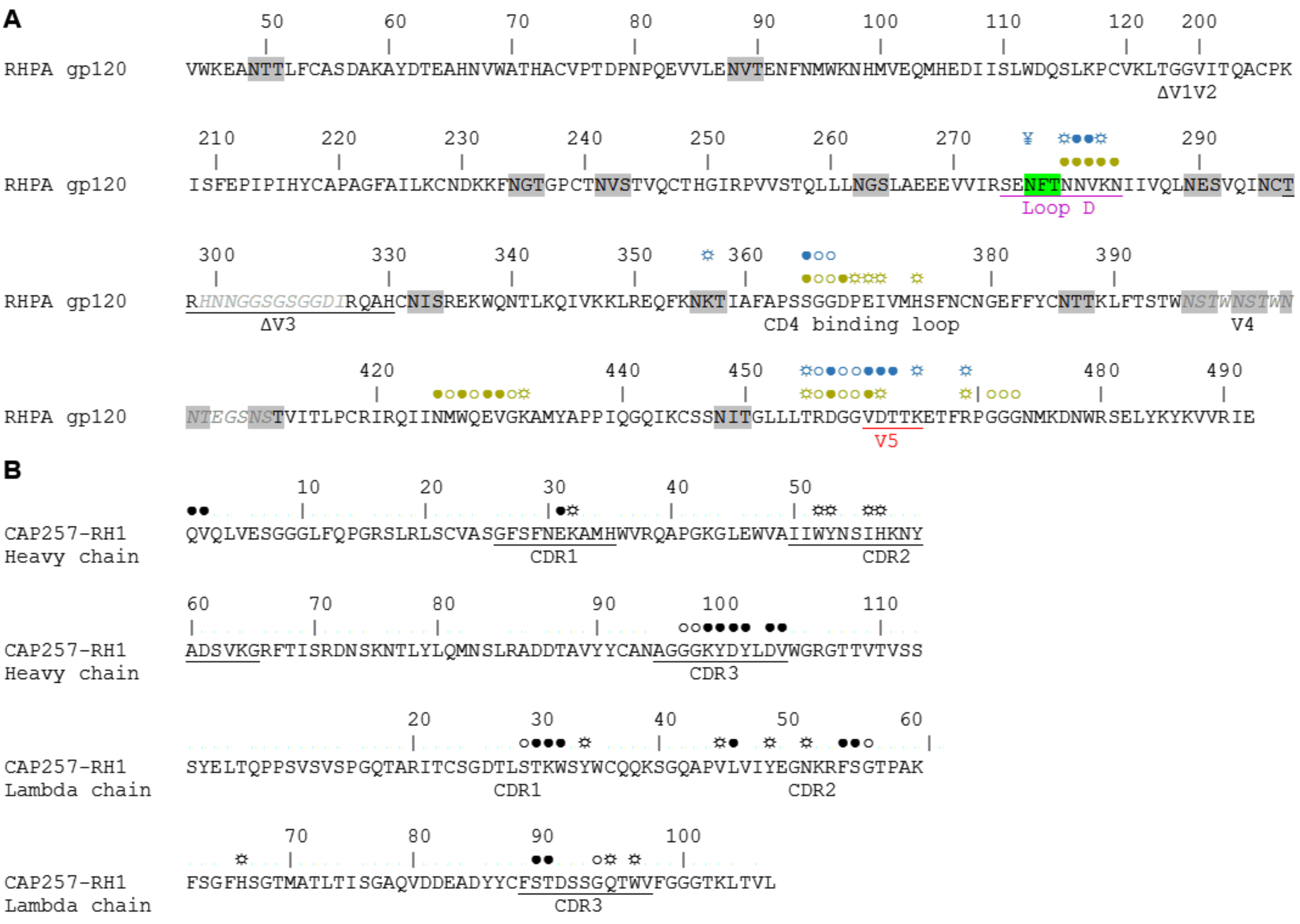

A) The RHPA gp120 sequence is shown, and numbered according to the HxB2 convention. Potential N-linked glycosylation sequons are shaded grey, disordered regions are indicated in italics, and loop D or V5 are indicate with purple and red underlines respectively. Contacts for the cocrystal complex are shown in blue, while previously determined interactions between CD4 and gp120 are shown in gold. Open circles (○) indicate main chain contacts only, ray circles (☼) indicate side chain contacts only, closed circles (●) indicate both main chain and side chain contacts, and the ¥ symbol is used to specify glycan interactions. B) The heavy and light chain sequences for CAP257-RH1 are shown, the CDR loops are underlined, and contacts in the cocrystal complex are labelled as in A.

Supplementary Figure 4: Autologous CAP257 V5 sequences

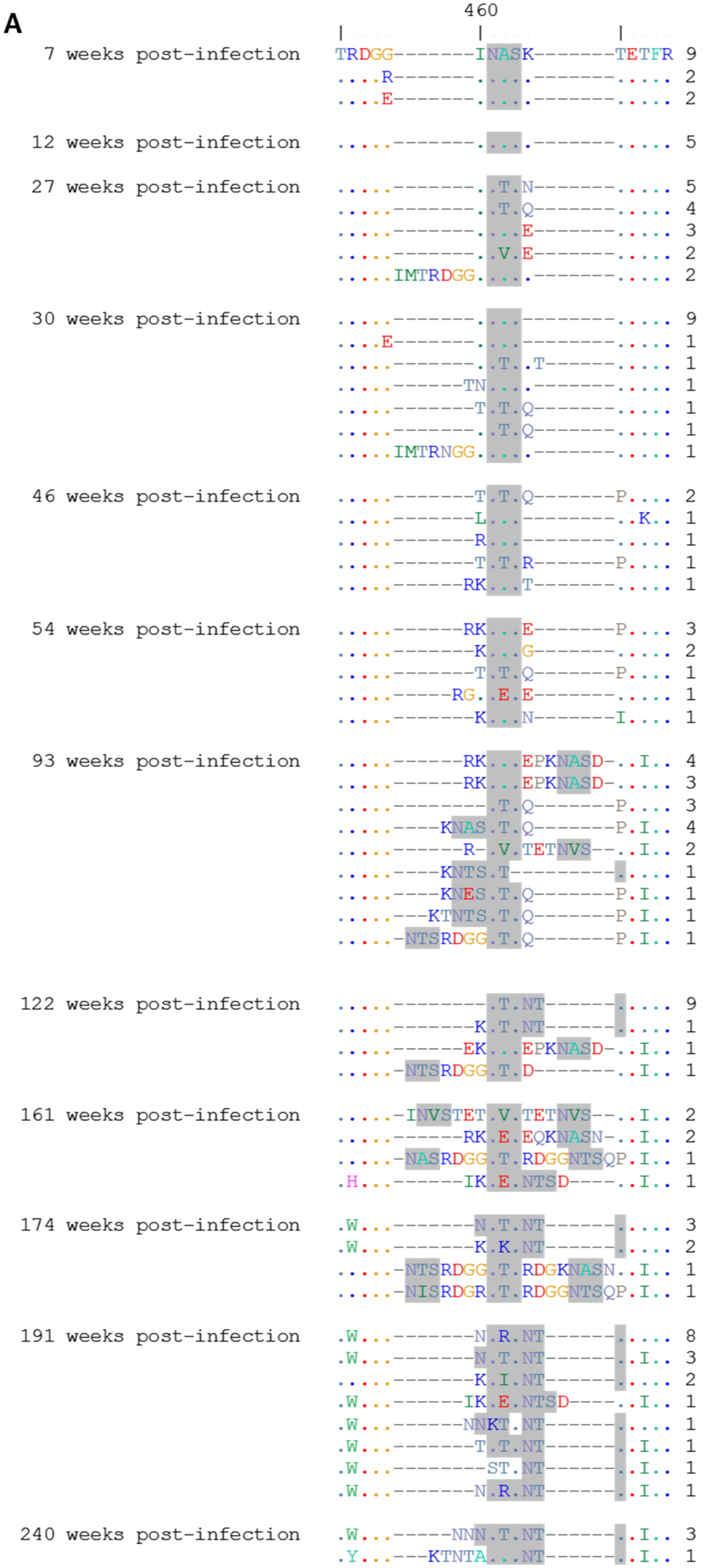

A) An alignment of CAP257 Env V5 (and flanking regions) sequences identified by SGA from 7 weeks to 240 weeks post-infection. Potential N-linked glycosylation sequons are shaded grey, and the number of identical sequences are listed on the right of the figure.

Supplementary Table 1: Heterologous neutralization mediated by CAP257-RH1

Virus	Clade	CAP257-RH1	Virus	Clade	CAP257-RH1	Virus	Clade	CAP257-RH1	Virus	Clade	CAP257-RH1
0260.v5.c36	A	>50	TH976.17	AE	>50	THRO.18	B	>50	CNE7	BC	>50
0330.v4.c3	A	>50	235-47	AG	>50	TRJO.58	B	>50	286.36	C	>50
0439.v5.c1	A	>50	242-14	AG	>50	TRO.11	B	>50	288.38	C	>50
3365.v2.c20	A	>50	263-8	AG	>50	WITO.33	B	>50	DU422.01	C	>50
3415.v1.c1	A	>50	269-12	AG	>50	X2278.C2.B1	B	>50	MW965.26	C	>50
3718.v3.c11	A	>50	271-11	AG	>50	YU2.DG	B	>50	SO18.18	C	>50
BB201.B42	A	>50	928-28	AG	>50	BJOX002000.03.2	BC	>50	TV1.29	C	>50
BI369.9A	A	>50	DJ263.8	AG	>50	CH038.12	BC	>50	TZA125.17	C	>50
BS208.B1	A	>50	T250-4	AG	>50	CH070.1	BC	>50	TZBD.02	C	>50
KER2008.12	A	>50	T251-18	AG	>50	CH117.4	BC	>50	ZA012.29	C	>50
KER2018.11	A	>50	T253-11	AG	>50	CH119.10	BC	>50	ZM106.9	C	>50
KNH1209.18	A	>50	T255-34	AG	>50	CH181.12	BC	>50	ZM109.4	C	>50
MB201.A1	A	>50	T257-31	AG	>50	CNE15	BC	>50	ZM135.10a	C	>50
MB539.2B7	A	>50	T266-60	AG	>50	CNE20	BC	>50	ZM176.66	C	>50
MI369.A5	A	>50	T278-50	AG	>50	CNE21	BC	>50	ZM197.7	C	>50
MS208.A1	A	>50	T280-5	AG	>50	CNE40	BC	>50	ZM214.15	C	>50
Q23.17	A	>50	T33-7	AG	>50	0013095-2.11	C	>50	ZM215.8	C	>50
Q259.17	A	>50	3988.25	B	>50	001428-2.42	C	>50	ZM233.6	C	>50
Q769.d22	A	>50	5768.04	B	>50	0077_V1.C16	C	>50	ZM249.1	C	>50
Q769.h5	A	>50	6101.10	B	>50	00836-2.5	C	>50	ZM53.12	C	>50
Q842.d12	A	>50	6535.3	B	>50	0921.V2.C14	C	>50	ZM55.28a	C	>50
QH209.14M.A2	A	>50	7165.18	B	>50	16055-2.3	C	>50	3326.V4.C3	CD	>50
RW020.2	A	>50	45_01dG5	B	>50	16845-2.22	C	>50	3337.V2.C6	CD	>50
UG037.8	A	>50	89.6.DG	B	>50	16936-2.21	C	>50	3817.v2.c59	CD	>50
246-F3.C10.2	AC	>50	AC10.29	B	>50	25710-2.43	C	>50	191821.E6.1	D	>50
3301.V1.C24	AC	>50	ADA.DG	B	>50	25711-2.4	C	>50	231965.c1	D	>50
3589.V1.C4	AC	>50	Bal.01	B	>50	25925-2.22	C	>50	247-23	D	>50
6540.v4.c1	AC	>50	BaL.26	B	>50	26191-2.48	C	>50	3016.v5.c45	D	>50
6545.V4.C1	AC	>50	BG1168.01	B	>50	3168.V4.C10	C	>50	57128.vrc15	D	>50
0815.V3.C3	ACD	>50	BL01.DG	B	>50	3637.V5.C3	C	>50	6405.v4.c34	D	>50
6095.V1.C10	ACD	>50	BR07.DG	B	>50	3873.V1.C24	C	>50	A03349M1.vrc4a	D	>50
3468.V1.C12	AD	>50	BX08.16	B	>50	6322.V4.C1	C	>50	NKU3006.ec1	D	>50
Q168.a2	AD	>50	CAAN.A2	B	>50	6471.V1.C16	C	>50	UG021.16	D	>50
Q461.e2	AD	>50	CNE10	B	>50	6631.V3.C10	C	>50	UG024.2	D	>50
620345.c1	AE	>50	CNE12	B	>50	6644.V2.C33	C	>50	P0402.c2.11	G	>50
BJOX009000.02.4	AE	>50	CNE14	B	>50	6785.V5.C14	C	>50	P1981.C5.3	G	>50
BJOX010000.06.2	AE	>50	CNE4	B	>50	6838.V1.C35	C	>50	X1193.c1	G	>50
BJOX025000.01.1	AE	>50	CNE57	B	>50	96ZM651.02	C	>50	X1254.c3	G	>50
BJOX028000.10.3	AE	>50	HO86.8	B	>50	BR025.9	C	>50	X1632.S2.B10	G	>50
C1080.c3	AE	>50	HT593.1	B	>50	CAP210.E8	C	>50	X2088.c9	G	>50
C2101.c1	AE	>50	HXB2.DG	B	>50	CAP244.D3	C	>50	SIVmac251.30.SG3	NA	>50
C4118.09	AE	>50	JRCSF.JB	B	>50	CAP45.G3	C	>50	SVA.MLV	NA	>50
CNE3	AE	>50	JRFL.JB	B	>50	Ce1176.A3	C	>50	Autologous TF:		
CNE5	AE	>50	MN.3	B	>50	CE703010217.B6	C	>50	CAP257.2.00.Luc	C	>50
CNE55	AE	>50	PVO.04	B	>50	CNE30	C	>50			
CNE56	AE	>50	QH0515.01	B	>50	CNE31	C	>50			
CNE59	AE	>50	QH0692.42	B	>50	CNE53	C	>50			
CNE8	AE	>50	REJO.67	B	>50	CNE58	C	>50			
M02138	AE	>50	RHPA.7	B	0.056	DU123.06	C	>50			
R1166.c1	AE	>50	SC422.8	B	>50	DU151.02	C	>50			
R2184.c4	AE	>50	SF162.LS	B	>50	DU156.12	C	>50			
TH966.8	AE	>50	SS1196.01	B	>50	DU172.17	C	>50			

Neutralization IC_{50} values of CAP257-RH1 tested on a multi-clade 196 virus panel. Titres <50 µg/mL are highlighted in red.

Supplementary Table 2: CAP257-RH1 interacting residues

	CDR/ FWR	Interfacial residue	Bond type*	Accessible Surface Area (Å^2)	Buried Surface Area (Å^2)	Buried Area (%)	$\Delta^i G$
Heavy chain	FW1	Gln 1	H	195.56	101.41	52 ||||||	-0.99
	FW1	Val 2		53.59	16.66	31 ||||	0.25
	H1	Glu 31	S	116.00	67.94	59 ||||||	-0.24
	H1	Lys 32		56.94	21.77	38 ||||	-0.72
	H2	Trp 52		33.50	9.12	27 |||	0.15
	H2	Tyr 52A		78.66	29.11	37 ||||	0.14
	H2	Ile 55		130.19	48.30	37 ||||	0.77
	H2	His 56		84.71	45.49	54 |||||	0.58
	H3	Gly 97		19.05	11.21	59 ||||||	0.18
	H3	Gly 98	H	11.81	11.81	100 ||||||||||	-0.04
	H3	Lys 99	HS	114.64	101.03	88 |||||||||	-1.06
	H3	Tyr 100		121.15	21.59	18 ||	-0.12
	H3	Asp 100A	H	50.24	35.83	69 |||||||	-0.42
	H3	Tyr 100B		152.86	16.67	11 |	0.07
	H3	Asp 101	H	79.99	35.73	45 |||||	-0.43
	H3	Val 102		45.90	27.26	59 ||||||	0.44
Light chain	L1	Leu 28		0.86	0.49	57 ||||||	-0.01
	L1	Ser 29		81.76	8.35	10 |	-0.10
	L1	Thr 30	H	101.81	54.99	54 |||||	-0.27
	L1	Lys 31	HS	51.09	37.28	73 |||||||	-0.24
	L1	Trp 32	H	110.50	64.92	59 ||||||	0.29
	L1	Tyr 34		41.75	20.81	50 |||||	-0.11
	FW2	Val 45		94.58	7.20	8 |	0.12
	FW2	Leu 46		72.34	13.00	18 ||	0.17
	L2	TYR 49		48.56	2.40	5 |	-0.02
	L2	Glu 50		45.55	4.06	9 |	-0.07
	L2	Lys 53		92.87	2.01	2 |	0.03
	L2	Phe 55		123.60	93.41	76 ||||||||	1.46
	L2	Ser 56		99.40	36.86	37 ||||	-0.40
	L2	GLY 57		78.97	26.61	34 |||	-0.19
	L2	THR 58		45.98	4.69	10 |	0.07
	FW3	SER 56		99.40	5.27	5 |	-0.06
	FW3	Gly 57		78.97	41.03	52 |||||	0.17
	FW3	His 66		44.46	5.93	13 |	-0.11
	L3	Ser 90		1.58	1.41	89 |||||||||	0.00
	L3	Thr 91		12.15	11.32	93 ||||||||||	0.07
	L3	Gly 95		36.71	2.51	7 |	0.04
	L3	Gln 95A		152.59	1.17	1 |	-0.01
	L3	Trp 96		146.62	14.16	10 |	0.23

FWR/CDR: Framework region or complementarity determining region of CAP257-RH1

*H = hydrogen bond, S = salt bridge.

Supplementary Table 3: RHPA gp120 interacting residues

gp120 region	Heavy chain interfacial residue	Bond type*	Accessible Surface Area (Å^2)	Buried Surface Area (Å^2)	Buried Area (%)	Δ^iG
Loop D	Asn 279		49.58	22.05	45 |||||	-0.32
	Asn 280	H	65.64	59.08	90 |||||||||	-0.54
	Val 281		109.40	90.06	82 |||||||||	1.29
	Lys 282	S	74.56	21.08	28 |||	0.15
CD4 binding loop	Ser 365		84.29	35.03	42 ||||	0.26
	Gly 366		57.60	18.67	32 |||	0.27
	Gly 367		57.76	27.64	48 |||||	0.20
β23	Thr 455		37.68	10.12	27 |||	0.14
	Arg 456	H	32.60	5.40	17 ||	-0.06
	Asp 457	HS	47.32	18.39	39 ||||	0.05
	Gly 458		22.15	5.58	25 |||	0.04
	Gly 459		73.12	41.61	57 ||||||	0.05
V5	Val 460		142.69	18.09	13 ||	0.29
β24	Arg 469		72.39	3.78	5		-0.04
Glycans	NAG1276		356.83	15.12	4		-0.28
	NAG1277		358.67	2.33	1		-0.03
	BMA1278		288.06	24.26	8		-0.30
	MAN1279	H	289.89	49.60	17 ||	-0.60
	MAN1280		289.41	14.44	5		0.32
	MAN1281	H	287.38	97.23	34 ||||	-0.93

gp120 region	Light chain interfacial residue	Bond type*	Accessible Surface Area (Å^2)	Buried Surface Area (Å^2)	Buried area (%)	Δ^iG
Loop E	Lys 354		190.51	2.50	1		-0.09
	Lys 357		68.78	8.09	12		-0.30
V5	Gly 459		73.12	10.59	15		-0.06
	Val 460	H	142.69	124.60	87 |||||||||	1.67
	Asp 461	HS	69.27	37.80	55 ||||||	-0.39
	Thr 462	H	121.42	96.85	80 ||||||||	0.79
	Thr 463		77.41	0.73	1		-0.01
	Lys 465		128.91	14.75	11		0.15
β24	Arg 469		72.39	1.02	1		-0.04
Glycans	NAG1277		358.67	43.42	12		-0.65
	BMA1278		288.06	16.07	6		-0.40
	MAN1280		289.41	73.16	25 |||	-1.17
	MAN1281		287.38	60.33	21 ||	-0.16
	MAN1282		290.50	45.77	16 ||	-0.57

*H = hydrogen bond, S = salt bridge.

Supplementary Table 4: List of hydrogen bonds and salt bridges

Hydrogen bonds

gp120 residue	Distance (Å)	Heavy chain residue
ASN 280[HD21]	2.39	ASP 100A[OD2]
ASN 280[OD1]	2.48	GLY 102 [H]
ARG 456[O]	1.81	LYS 99 [HZ1]
ASP 457[OD1]	2.40	LYS 99 [HZ2]
MAN1279[O5]	2.28	GLN 1 [HE21]
MAN1281[O6]	2.40	ASP 108 [OD2]
**gp120 residue**	**Distance (Å)**	**Light chain residue**
VAL 460[O]	2.13	TRP 32[H]
ASP 461[OD1]	2.16	LYS 31[HZ3]
THR 462[H]	2.34	THR 30[O]

Salt bridges

gp120 residue	Distance (Å)	Heavy chain residue
LYS 282[NZ]	3.58	GLU 31[OE2]
ASP 457[OD1]	3.20	LYS 99[NZ]
**gp120 residue**	**Distance (Å)**	**Light chain residue**
ASP 461[OD1]	3.04	LYS 31[NZ]

Supplementary Table 5: Frequency of glycosylation at position N276 and in the V5 loop of a multi-clade 196 virus panel

Virus	N276	V5 Sequence	Virus	N276	V5 Sequence	Virus	N276	V5 Sequence	Virus	N276	V5 Sequence
0260.v5.c36	Y	DNNNTTT	TH976.17	Y	ANNSAS	THRO.18	Y	SDGGSKNSSKNETGT	CNE7	N	INGTNNT
0330.v4.c3	Y	NNNNGANGN	235-47	Y	GNSSSAN	TRJO.58	Y	KTANNTT	286.36	Y	IINDT
0439.v5.c1	Y	NNATN	242-14	Y	FRNDTNETY	TRO.11	Y	NNNSSGP	288.38	Y	TNNTNNT
3365.v2.c20	Y	NNNNSSN	263-8	Y	INNSTS	WITO.33	Y	SNSSQN	DU422.01	Y	ENSTE
3415.v1.c1	Y	ENNST	269-12	Y	LNDSSTN	X2278.C2.B1	Y	ENNGTT	MW965.26	Y	ENNRT
3718.v3.c11	Y	ENET	271-11	Y	NNNNNAT	YU2.DG	Y	KDTNGT	SO18.18	Y	HNSTN
BB201.B42	Y	NNNGTS	928-28	Y	NNGTNDT	BJOX002000.03.2	Y	PENDT	TV1.29	Y	FNTTNNT
BI369.9A	Y	GNNNTN	DJ263.8	N	SNNSTN	CH038.12	Y	RSNETNDT	TZA125.17	Y	NNTNGT
BS208.B1	Y	YNNTN	T250-4	N	NSTQN	CH070.1	Y	NINRTN	TZBD.02	Y	GESNET
KER2008.12	Y	NSTVN	T251-18	Y	NNGSKAN	CH117.4	Y	RDINNT	ZA012.29	Y	EDNKTE
KER2018.11	Y	NTGNNSRTN	T253-11	Y	ANSSTN	CH119.10	Y	TESNNTETNNT	ZM106.9	Y	NGDTNDT
KNH1209.18	Y	NGNSTN	T255-34	Y	NNNSMN	CH181.12	Y	ETNQTNFT	ZM109.4	Y	NNNNSTE
MB201.A1	Y	NNNGTS	T257-31	Y	DSNDTNGN	CNE15	Y	NEDKNNT	ZM135.10a	Y	LNNKENGT
MB539.2B7	Y	NDNSTNEN	T266-60	Y	VNNDTNTT	CNE20	Y	TESNDT	ZM176.66	Y	NDDNDT
MI369.A5	Y	GNNNTN	T278-50	Y	GDEKAN	CNE21	Y	MTNETNKT	ZM197.7	Y	NKSAGI
MS208.A1	Y	PNTTN	T280-5	N	NNSGSN	CNE40	Y	KEANGT	ZM214.15	Y	NGNDTNDT
Q23.17	Y	KDNNVN	T33-7	Y	NNNNAT	0013095-2.11	Y	NGNDTNKT	ZM215.8	Y	TNNTN
Q259.17	Y	KDSNIT	3988.25	Y	NNNNTNTT	001428-2.42	Y	KNNNT	ZM233.6	Y	ENSSSTT
Q769.d22	Y	IINSTE	5768.04	Y	GSNSSNTSE	0077_V1.C16	Y	ETNDGNTNETGTKTFKSNETET	ZM249.1	Y	SKNNTE
Q769.h5	Y	IINSTDTD	6101.1	Y	DNNNTI	00836-2.5	N	NHEEANTT	ZM53.12	Y	LTNESK
Q842.d12	Y	NTNSTR	6535.3	Y	NETNVT	0921.V2.C14	Y	NGGADNST	ZM55.28a	Y	ENTTDNGT
QH209.14M.A2	Y	DDENNT	7165.18	Y	ENRTDNGT	16055-2.3	Y	VESNET	3326.V4.C3	Y	NSH
RW020.2	Y	NNSTN	45_01dG5	N	SSTNGTT	16845-2.22	Y	NSNDTNEP	3337.V2.C6	Y	NTSE
UG037.8	Y	GNINES	89.6.DG	Y	NSTETET	16936-2.21	Y	PDNVT	3817.v2.c59	Y	LNTSNN
246-F3.C10.2	Y	NTNSTK	AC10.29	Y	RGNQTDNQT	25710-2.43	Y	TGSESNKT	191821.E6.1	Y	INSNTSSS
3301.V1.C24	Y	SDGNSTK	ADA.DG	Y	TNSSGS	25711-2.4	Y	RGEEVKNDT	231965.c1	Y	RDNSSSN
3589.V1.C4	Y	GNNSSEN	Bal.01	Y	PEDNKT	25925-2.22	Y	TGLNSST	247-23	N	SNNTNET
6540.v4.c1	N	NNNSDN	BaL.26	Y	PEDDKT	26191-2.48	Y	NSNSTE	3016.v5.c45	Y	NTSDDH
6545.V4.C1	N	NRSSDN	BG1168.01	Y	DTNNGT	3168.V4.C10	Y	KGNNET	57128.vrc15	Y	GADNNRQN
0815.V3.C3	Y	SNTNAS	BL01.DG	Y	KNGTEGT	3637.V5.C3	Y	ISNGTDNKN	6405.v4.c34	Y	ADNSSHN
6095.V1.C10	Y	VNNDSQS	BR07.DG	Y	NTSSEA	3873.V1.C24	Y	TGKNDTENAT	A03349M1.vrc4a	Y	GNESSQN
3468.V1.C12	Y	DTSSMN	BX08.16	Y	SDNSSSGK	6322.V4.C1	Y	KDNNMT	NKU3006.ec1	Y	NTNSTN
Q168.a2	Y	NNNSTN	CAAN.A2	Y	NDNETNGT	6471.V1.C16	N	KTSNDPDT	UG021.16	Y	VKNNSQN
Q461.e2	Y	GENDT	CNE10	Y	NESTTTET	6631.V3.C10	Y	PNSTN	UG024.2	Y	NTSQN
620345.c1	N	GGPTADN	CNE12	Y	KNESNTT	6644.V2.C33	Y	SINGSEDENNT	P0402.c2.11	Y	GNNTNET
BJOX009000.02.4	Y	DINASN	CNE14	Y	DNENTTT	6785.V5.C14	Y	SINGSEDNT	P1981.C5.3	Y	NTNGT
BJOX010000.06.2	Y	NISATN	CNE4	Y	HNGNGSNET	6838.V1.C35	Y	NGTERN	X1193.c1	Y	KSNETNGT
BJOX025000.01.1	N	NNTNTS	CNE57	Y	NNTDKNT	96ZM651.02	Y	STNDSTNNNT	X1254.c3	Y	TNNNTN
BJOX028000.10.3	Y	DDNGT	HO86.8	Y	KNNKST	BR025.9	Y	TGMHDT	X1632.S2.B10	Y	TNKDTNEA
C1080.c3	Y	DANATNDT	HT593.1	Y	NNNKTNGT	CAP210.E8	Y	ENKTENNDT	X2088.c9	Y	NDTHDKEN
C2101.c1	Y	NNNTN	HXB2.DG	Y	NSNNES	CAP244.D3	Y	EANGTNNI	SIVmac251.30.SG3	N/A	-
C4118.09	Y	TNNNTNSS	JRCSF.JB	Y	KNESEI	CAP45.G3	Y	KTDRNDT	SVA.MLV	N/A	-
CNE3	N	NNSNAN	JRFL.JB	Y	INENGT	Ce1176.A3	Y	INQTGE	Autologous T/F:		
CNE5	Y	NNNSTGN	MN.3	Y	EDTDTNDT	CE703010217.B6	Y	NNKSTP	CAP257.2.00.Luc	Y	INASK
CNE55	Y	GNNRSN	PVO.04	Y	ANNTN	CNE30	Y	TTNNTNNT			
CNE56	Y	DNTTDS	QH0515.01	Y	TNGTNET	CNE31	Y	KTNKTE			
CNE59	Y	NNMTN	QH0692.42	Y	VNGTR	CNE53	Y	SQPNNT			
CNE8	Y	TNMSN	REJO.67	Y	NSSLSSP	CNE58	Y	TNNATT			
M02138	Y	INRTYN	RHPA.7	Y	VDTTK	DU123.06	Y	NTSNSTP			
R1166.c1	Y	VNNSTN	SC422.8	Y	NNNSTT	DU151.02	Y	KNTTN			
R2184.c4	Y	TNTSKNN	SF162.LS	Y	KEISNTT	DU156.12	Y	GNVTEINRT			
TH966.8	Y	ANNTSN	SS1196.01	Y	NNTTTNGTQT	DU172.17	Y	KEKNDT			

The V5 loop sequence motifs for the CAP257 transmitted/founder clone, and the 196 heterologous strain from supplementary table 1 are shown. Potential N-linked glycosylation sequons are highlighted in red. The presence or absence of the N276 glycan for each virus is indicated.
